# Accelerometry-measured prolonged and interrupted sedentary behavior and cancer incidence and mortality: A cohort study of 91,292 UK Biobank participants

**DOI:** 10.1371/journal.pmed.1004767

**Published:** 2026-07-02

**Authors:** Ziyi Zhou, Stewart G. Trost, Gemma C. Ryde, Solange Parra-Soto, Zhe Fang, Chao Xu, Yujia Lu, Kai Wang, Mengxi Du, Zhi Li, Yuebin Lv, Jason M.R. Gill, Stuart R. Gray, Carlos Celis-Morales, Marc J. Gunter, Edward Giovannucci, Jill P. Pell, Mingyang Song, Frederick K. Ho

**Affiliations:** 1 School of Health and Wellbeing, University of Glasgow, Glasgow, United Kingdom; 2 Department of Epidemiology, Harvard T.H. Chan School of Public Health, Boston, Massachusetts, United States of America; 3 Clinical and Translational Epidemiology Unit, Massachusetts General Hospital and Harvard Medical School, Boston, Massachusetts, United States of America; 4 School of Human Movement and Nutrition Sciences, The University of Queensland, Brisbane, Australia; 5 School of Cardiovascular and Metabolic Health, University of Glasgow, Glasgow, United Kingdom; 6 Department of Nutrition and Public Health, Universidad del Bío-Bío, Chillan, Chile; 7 China CDC Key Laboratory of Environment and Population Health, National Institute of Environmental Health, Chinese Center for Disease Control and Prevention, Beijing, China; 8 Human Performance Laboratory, Education, Physical Activity and Health Research Unit, Universidad Catolica del Maule, Talca, Chile; 9 High-Altitude Medicine Research Centre (CEIMA), Universidad Arturo Prat, Iquique, Chile; 10 Nutrition and Metabolism Branch, International Agency for Research on Cancer, World Health Organization, Lyon, France; 11 Department of Epidemiology and Biostatistics, School of Public Health, Imperial College London, London, United Kingdom; 12 Department of Nutrition, Harvard T.H. Chan School of Public Health, Boston, Massachusetts, United States of America; 13 Division of Gastroenterology, Massachusetts General Hospital and Harvard Medical School, Boston, Massachusetts, United States of America; 14 Microbiome Lab, Broad Institute of MIT and Harvard, Cambridge, Massachusetts, United States of America; National Cancer Institute, UNITED STATES OF AMERICA

## Abstract

**Background:**

Current sedentary behavior (SB) guidelines primarily emphasize total time spent sedentary. We explored differences between interrupted and prolonged SB in relation to a range of cancer outcomes.

**Methods and findings:**

This study included 91,292 UK Biobank participants with valid accelerometer data. Participants were followed for a median of 12.38 years (interquartile range 11.56–13.15 years). A two-step approach based on a random forest model was used to classify SB. Multivariable Cox proportional hazards models were applied to overall incident cancers and cancer deaths, plus obesity-related and type-2 diabetes-related cancers, and 23 site-specific cancers. Models were adjusted for demographic, socioeconomic, lifestyle, dietary, and health-status factors, including age, ethnicity, deprivation, education, smoking, alcohol intake, diet, and morbidity count. Isotemporal substitution models were used to estimate the associated cancer risk when replacing prolonged SB with intermittent SB, or physical activity (PA). After adjusting for sociodemographic and lifestyle factors, each additional hour of prolonged SB was associated with a higher risk of overall cancer mortality (hazard ratio [HR] HR_1hour_ 1.09; 95% confidence interval [CI] [1.06, 1.11]; *p* < 0.001). Replacing 1 hour per day of prolonged SB with light PA (HR_LPA_ 0.88; 95% CI [0.79, 0.99]; *p* = 0.033) was associated with lower risk of overall cancer mortality. Similarly, replacing 30 min per day of prolonged SB with moderate PA (HR_MPA_ 0.92; 95% CI [0.86, 0.99]; *p* = 0.024) was associated with a lower risk of overall cancer mortality. The main methodological limitations were observational design, residual confounding, healthy volunteer bias, and measurement imprecision due to having only 7 days of accelerometer wear.

**Conclusion:**

Cancer risk associated with SB is specific to prolonged SB. Replacing prolonged SB physical activity is associated with lower cancer risk.

## Author summary

**Why was this study done?:**

- Previous studies have shown that spending more total time on sedentary behavior, such as sitting or reclining while awake, is linked to poorer health outcomes.
- Some evidence suggests that replacing sedentary time with physical activity (PA) may lower the risk of cancer death, but less is known about whether the pattern of sedentary time matters.
- This study was done to examine whether long, uninterrupted periods of sedentary behavior and shorter, interrupted periods of sedentary behavior are differently associated with cancer risk.

**What did the researchers do and find?:**

- We studied 91,292 UK Biobank participants with valid activity monitor data and followed them for a median of 12.38 years.
- Each additional hour per day of prolonged sedentary behavior was associated with a 10% higher hazard of cancer death, whereas each additional hour of interrupted sedentary behavior was associated with a 19% lower hazard of cancer death.
- Replacing prolonged sedentary behavior with PA was associated with a lower risk of cancer death: a 12% lower hazard when 1 hour per day was replaced with light PA, an 8% lower hazard when 30 min per day was replaced with moderate PA, and 22% lower hazard when 5 min per day was replaced with vigorous PA.

**What do these findings mean?:**

- These findings suggest that not only the total amount of sedentary time, but also how sedentary time is accumulated, may be important for cancer risk.
- Reducing long, uninterrupted periods of sedentary behavior and replacing them with PA, even those with light intensity, may be a practical target for future interventions.
- These findings should be interpreted with caution because the study cannot prove causality, UK Biobank volunteers may not represent the wider population, and the activity monitor captured behavior only during a limited period without showing the context of sedentary behavior, such as work, television viewing, or driving.

## Abstract 3

In a prospective cohort study, Ziyi Zhou and colleagues use a machine learning approach to investigate the association between prolonged and interrupted sedentary behavior in relation to cancer outcomes in 92,354 UK Biobank participants.

## Introduction

Sedentary behavior (SB), typically defined as any waking activities involving an energy expenditure of 1.5 metabolic equivalents of task (METs) or less while in a sitting, reclining, or lying posture, accounts for ~55% of waking time in both children and adults based on self-reported data [1]. Existing evidence consistently demonstrates that excessive SB, such as prolonged daily sitting, is a risk factor for cardiovascular disease (CVD), type-2 diabetes (T2D), obesity, certain cancers, and all-cause mortality [2,3], particularly among individuals with low levels of physical activity (PA) [4]. This association may result from SB replacing PA or as an independent effect.

Recent evidence suggests that how SB is accumulated is important; not only the total amount of SB accumulated [1]. Prolonged, uninterrupted bouts of SB have been identified as potentially the most hazardous and interrupting prolonged bouts with activity breaks has been recommended by several health agencies as a viable strategy to mitigate associated health risks [5]. Experimental crossover studies provide a plausible biological rationale for this recommendation: interrupting prolonged sitting with brief bouts of light- or moderate-intensity activity attenuates postprandial glucose and insulin responses in both overweight/obese and healthy normal-weight adults, indicating that the pattern of SB accumulation can acutely influence metabolic regulation [6,7]. However, current evidence regarding optimal frequency and duration of sedentary breaks remains limited; it is often based on self-reported data, and experimental studies often lack clinical endpoints [8]. Importantly, the roles of interrupted and prolonged SB in cancer outcomes have not been examined.

In addition to examining whether types of SB could be related to cancer risks, it is also important to examine how best to change or replace it. It is not possible to reduce SB without increasing the time spent on other activities. Potential harms associated with SB may be mitigated through a replacement with PA [1,9]. According to the *2018 Physical Activity Guidelines Advisory Committee Scientific Report* [1], higher amount of moderate to vigorous PA (e.g., 40–100 min per day or over 300 min per week) may offset or eliminate the risks associated with prolonged SB. It is also possible that risk could be lowered simply by interrupting SB with some forms of PA without actually increasing the total PA. However, there is a lack of evidence on these important questions in relation to cancer outcomes, especially based on objective measurements of SB and PA.

In addition, it is also important to examine whether the association of SB with cancer differs by obesity status [10,11]. Obesity is an important risk factor for cancer, and previous studies have shown obesity to modify the associations between SB and PA and health outcomes [12,13]. If the cancer risk associated with SB differs between people with and without obesity, interventions may need to be tailored to optimize health outcomes across different population subgroups.

To address these gaps, we conducted a prospective cohort study to examine whether prolonged and interrupted sedentary behavior are differently associated with cancer risk. Specifically, we evaluated associations of prolonged and interrupted sedentary behavior with overall incident cancer, overall cancer mortality, obesity-related cancers, T2D-related cancers, and 23 site-specific cancers. We also examined whether replacing time spent in sedentary behavior with other daily behaviors, including PA, was associated with cancer risk, and whether associations differed by obesity status. We hypothesized that prolonged sedentary behavior would be associated with higher cancer risk, whereas interrupted sedentary behavior and replacing sedentary time with PA would be associated with lower cancer risk.

## Methods

UK Biobank is a prospective, population-based cohort study that recruited 502,493 participants aged 37–73 years at baseline from the general population (with a 5.5% response rate) between 2006 and 2010 [14]. Participants attended one of 22 assessment centers across Scotland, England, and Wales [14]. All participants completed touch-screen questionnaires and underwent physical measurements related to social demography, lifestyle, health, and physical assessments at baseline [15]. More information about the UK Biobank protocol can be found online (http://www.ukbiobank.ac.uk).

**Fig 1:**
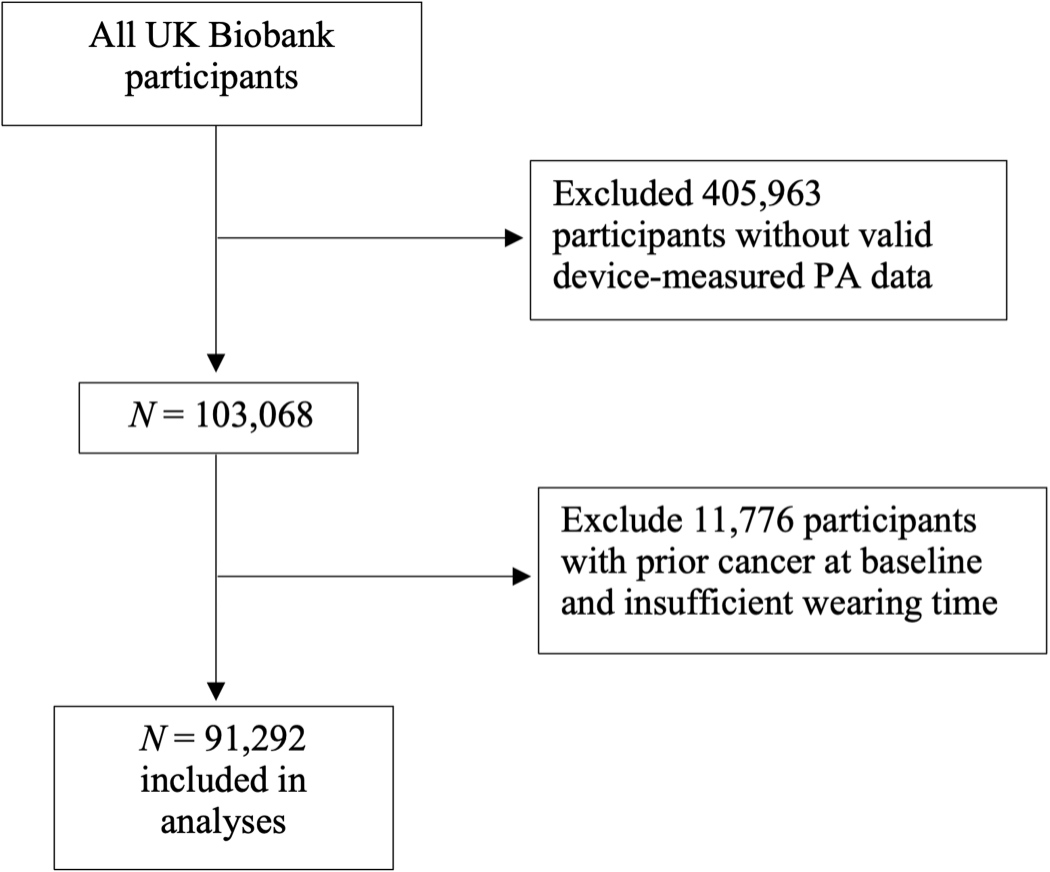
Participants flowchart.

Of the 103,068 participants with valid device-measured PA data, 11,776 were excluded because they had a history of cancer at baseline, had insufficient wearing time (<72 hours), or withdrew from UK Biobank. A total of 91,292 UK Biobank participants were included in this study (Fig 1). The median (interquartile range) of follow-up was 12.38 (11.56–13.15) years. This study is reported as per the Strengthening the Reporting of Observational Studies in Epidemiology (STROBE) guideline (S1 Checklist).

### Accelerometer-measured PA and sedentary behaviors

Accelerometer-measured PA was obtained, using Axivity AX3 wrist-worn triaxial accelerometers, from 103,567 UK Biobank participants between 2013 and 2015. Participants who provided an email address to UK Biobank were invited at random [16]. The accelerometer was worn on the dominant wrist for a period of 7 days, with recording at 100 Hz, as has been described elsewhere [16]. The accelerometer is water resistant up to 1.5 meters and the participants were advised to wear it for 7 days continuously. Only participants in the accelerometer sub-study were included in this analysis. Those with insufficient wear time (*n* = 8,480; < 96 hours, based on GGIR rule) or poor device calibration (*n* = 11; based on previous UK Biobank analysis [16]) were excluded. There were 95,076 participants with valid accelerometer data.

A validated two-step classifier based on a random forest model was used to categorize raw acceleration as SB, light, moderate and vigorous PA [17,18]. Previous field evaluations showed positive and negative predictive values for sedentary behavior to be 0.88 and 0.97, respectively [19]. Briefly, the random forest model uses features in the raw acceleration signal to classify raw accelerometer data (100 Hz) into 10-s window of SB (e.g., watching television), utilitarian movements (e.g., ironing a shirt, washing dishes), walking, or high energy activities. Light PA (LPA) included utilitarian movements, and walking at a low speed (<3mph); moderate PA (MPA) included walking activities at a moderate speed (3–4 mph); vigorous PA (VPA) included walking activities at brisk intensity (>4 mph) and high energy activities (e.g., running, actively playing a tennis game). It should be noted that accelerometry provided precise measurements of PA intensity in 10-s intervals as opposed to self-reported measurements, which provide average measurements for given activities. For example, playing badminton leisurely is usually classified as MPA based on self-reported data. However, it actually consists of standing with movement, walking, and running, and the classifier would thus identify a mix of these activity classes over the duration of game play. Mapping these to the traditional intensity-based categories, a combination of LPA, MPA, and VPA would be identified, whether the ‘average intensity’ would depend on the proportion of those PA. This two-step classifier was found to be more accurate than classification purely based on accelerations [17,18], and has been previously used and published [20,21]. Sleep was classified based on a validated method [22] to distinguish nonwear from sleep.

Prolonged SB was defined as any bout of SB that lasted at least 30 min and during which at least 90% of the time was sedentary, while interrupted behaviors to be those that did not meet the threshold above, i.e., those lasted less than 30 min or those broken down by >10% of non-sedentary behaviors. Prolonged and interrupted SB are mutually exclusive. We defined prolonged SB different from PAs to better capture how individuals allocate their time and to coincide with existing evidence. Current randomized controlled trial (RCT) and cohort studies indicate often defined prolonged sedentary bouts as those ≥ 30 min [23–25]. The accelerometer data scoring was done using the publicly available R package actimetric (https://github.com/PhysicalActivityOpenTools/actimetric) with the ‘Adult Wrist RF Trost’ algorithm.

### Outcomes

The study outcomes were overall cancer mortality and incidence of overall, obesity-related, T2D-related, and site-specific cancers. After excluding cancers with case numbers fewer than 50, 17 cancer sites were included for both sexes (esophageal, kidney, stomach, liver, pancreas, colorectal, bladder, oral, lung, melanoma, thyroid, multiple myeloma and malignant plasma cell neoplasms, brain, gallbladder, leukemia, non-Hodgkin lymphoma and hepatocellular carcinoma), two specific to men (testicular and prostate cancer), and four specific to women (breast, uterine, cervical, and ovary cancer). Obesity-related and T2D-related cancers were identified based on strong or highly suggestive existing evidence of their relationships with obesity and T2D, respectively [26]. Specifically, obesity-related cancers included esophageal, liver, kidney, myeloma, pancreatic, colorectal, gallbladder, breast, ovarian, and thyroid cancer. T2D-related cancers included thyroid, breast, liver, pancreatic, endometrial, esophageal, colorectal, kidney, gallbladder, ovarian, bladder cancer, and non-Hodgkin lymphoma and leukemia.

Participants provided consent for UK Biobank to follow their health over time through linkage to electronic medical and other health-related records [27]. Mortality data were available up to November 2022 and hospital admission data were available up to October 2022 in England, August 2022 in Scotland, and May 2022 in Wales. We used the International Classification of Diseases, 10th revision (ICD-10), to ascertain cancer diagnoses: overall cancer (C00–C97, D37, D48), oral (lip, pharynx and larynx) (C00–C14), esophageal (C15), colorectal (C18, C19, and C20), liver (C22), pancreas (C25), lung (C34), melanoma (C43), breast (C50), uterine (C54–C55), ovarian (C56), prostate (C61), kidney (C64-C65), bladder (C67), brain (C71), non-Hodgkin lymphoma (C82–C85), multiple myeloma and malignant plasma cell neoplasms (C90; 93% multiple myeloma), and leukemia (C91–C95).

### Obesity measures

Body mass index (BMI) was calculated as weight (kg) divided by height squared (m²). Standing height (cm) was measured using a Seca 202 device (SECA). Weight was measured using the Tanita BC-418MA body composition analyzer (Tanita Corporation of America). Waist and hip circumferences were measured in centimeters by trained nurses following a standardized protocol. Wessex nonstretchable sprung tape was used to measure waist and hip circumferences [28], and the waist-to-hip ratio (WHR) was calculated by dividing waist circumference by hip circumference [29]. Waist-to-height ratio (WHtR) was calculated by dividing waist circumference by height [29]. General obesity was defined as a BMI ≥ 30 kg/m². Central obesity was classified as follows: WHR > 0.95 for men and >0.85 for women; waist circumference (WC) ≥ 102 cm for men and ≥ 88 cm for women; and WHtR > 0.5 [29].

### Covariates

Participants’ age at the time of PA data collection was calculated using their date of birth and the date of data collection. Ethnicity was self-identified and grouped into categories: White, South Asian, Black, Chinese, other, or mixed. Socioeconomic deprivation was assessed using area-based continuous Townsend scores derived from participants’ postcodes at baseline [30]. Educational level was determined from the highest qualification reported by participants. Smoking status was self-reported and categorized as never, former, or current smoker. Alcohol consumption was calculated based on self-reported frequency and amount of alcohol intake. Dietary intake, including fruit and vegetables, red meat, processed meat, and oily fish, was assessed through a baseline food frequency questionnaire completed by participants. Morbidity count was assessed by summing 43 self-reported long-term conditions (LTCs), with participants classified as having multiple long-term conditions (MLTCs) if they reported two or more. The list of the 43 conditions is in Table A of S1 Appendix and has been used before in UK Biobank [31,32].

### Statistical analyses

The characteristics of participants stratified by quartiles of total, prolonged, and interrupted SB, were summarized using means (standard deviations [SDs]) and frequencies (percentages) for quantitative and categorical variables, respectively. Cox proportional hazards models were used to analyze associations between total, prolonged, and interrupted SB and cancer outcomes, including overall cancer mortality; overall, obesity-related, and T2D-related incident cancer; as well as 23 sites-specific incident cancers. Note that even though the Cox model technically examines hazard (an instantaneous incidence rate), the associations were interpreted as ‘risk’ for easier reading. This approximation should be valid given that the outcomes are rare and that the direction of association between risk and hazard should be consistent.

Two multivariable models were used for each outcome: Model 1 adjusted for age, sex, and ethnicity; Model 2 additionally adjusted for deprivation, education, smoking status, and intake of alcohol, sugar, processed meat, red meat, fruit and vegetables, and oily fish. These covariates were selected as they were potential confounders. Sensitivity analyses further adjusting for BMI and morbidity count were conducted to examine the associations conservatively, since BMI and morbidity count could be confounders or mediators.

Cox proportional hazards models were also applied to analyze associations between different intensities of PA and composite and site-specific cancers. SB-adiposity interactions were additionally assessed by including a multiplicative interaction term in the model. Penalized cubic spline models were used to assess potential nonlinear relationships between each type of SB, PA and the three composite cancer outcomes.

Isotemporal substitution models (ISM) were used to estimate associated change in risk of replacing a specified amount of type-specific SB with equivalent durations of other activities [33]. Two separate ISMs were fitted. In the first ISM, prolonged SB was treated as the activity being replaced. This model estimated the associated change in risk when replacing prolonged SB with an equal duration of another behavior. For example, the coefficient for interrupted SB indicates the change in risk when 1 hour of prolonged SB was replaced by 1 hour of interrupted SB, holding all other behaviors unchanged. Similarly, the coefficients for LPA, MPA, and VPA indicate the associated risk when 1 hour, 30-min, and 5-min of LPA, MPA, and VPA were used to replace the equal duration of prolonged PA. These durations were selected based on the approximate interquartile range of each activity. In the second ISM, interrupted SB was treated as the activity and prolonged SB being replaced and the specification is similar. The formulae for the two ISMs were as follows:

Model 1 (replacing prolonged SB):

*h(t)* = *h*_*0*_*(t)* * exp (β_1_ sleep + β_2_ LPA + β_3_ MPA + β_4_ VPA + β_5_ interrupted SB + β_k_ Covariates)

Model 2 (replacing interrupted SB):

*h(t)* = *h*_*0*_*(t)* * exp (β_1_ sleep + β_2_ LPA + β_3_ MPA + β_4_ VPA + β_5_ prolonged SB + β_k_ Covariates)

where *h(t)* is the hazard function and *h*_*0*_*(t)* is the baseline hazard, and all behavioral variables were continuous variables representing the individual’s time spent in those behaviors. Due to the perfect multicollinearity inherent in 24-hour behavioral data, where the sum of time spent on sleep, prolonged SB, interrupted SB, LPA, MPA, and VPA must be 24 hours, one behavior was omitted from each model to act as the reference. In this framework, the coefficients of the included behaviors represent the theoretical effect of increasing that behavior by one unit while simultaneously decreasing the omitted behavior by the same amount. For instance, in Model 1, β_2_ represents the estimated hazard ratio for replacing one unit of prolonged SB with LPA. In this paper, only β_2 –_ β_5_ were reported because sleep duration could be confounded by mental health and is outside the scope of this study. The ISM analyses were conducted adjusted for deprivation, education, smoking status, and intake of alcohol, sugar, processed meat, red meat, fruit and vegetables, and oily fish. All analyses were conducted using R version 4.0.3. There was no prospective analysis protocol for this study and there were no changes to the analysis plan. The data was re-analyzed once during the peer review process due to a significant update to the actimetric package used to ascertain SB and PA.

### Ethics and consent

This study was conducted using data from the UK Biobank Resource under Application Numbers 71392. These application numbers refer to approvals granted by UK Biobank for access to and use of the data. UK Biobank has ethical approval from the Northwest Multi-centre Research Ethics Committee (REC reference: 11/NW/03820), and all participants provided written informed consent for participation and follow-up through linkage to health-related records.

## Results

There were 91,292 participants included in the final analysis (Tables 1 and Tables A–D in S1 Appendix). Of these, 51,169 [56.0%] were female and the mean age was 56.0 years (interquartile range: 7.4–7.8 years). The mean age of participants increased progressively across quartiles of total sedentary time from 54.0 years in Q1 to 58.2 years in Q4. Participants with higher total sedentary time were more likely to be deprived: mean = −1.9 [SD 2.7] in Q1 versus mean = −1.4 [SD 3.0] in Q4 and report higher educational levels. Higher total sedentary time was associated with increased weekly intake of red meat (2.0 times in Q1 versus 2.1 times in Q4) and processed meat (1.3 times in Q1 versus 1.5 times in Q4). Compared with total SB, similar findings were found across prolonged SB quartiles (Table C in S1 Appendix). Participants with prolonged SB were also more likely to be deprived - mean = −1.8 [SD 2.8] in Q1 versus mean = −1.5 [SD 3.0] in Q4 and consumed red meat more frequently, from 2.0 to 2.1 per week across the quartiles. On the contrary, compared with both total and prolonged SB, participants with higher amounts of interrupted SB had lower red meat intake, from 2.1 to 2.0 times per week and higher fruits and vegetable intake from 4.0 to 4.3 serving per day for Q1 and Q4, respectively (Table D in S1 Appendix). The proportion of participants experiencing cancer mortality, and overall, obesity-related, and T2D-related incident cancers incidence was higher in Q4 of SB than in Q1. Similar findings were also observed for several site-specific cancers: esophageal, kidney, liver, colorectal, lung, and prostate cancer (Table B in S1 Appendix).

**Table 1:** Baseline characteristics of participants by total sedentary behavior quartiles. Values are presented as mean ± standard deviation or number (%), unless otherwise stated.

Characteristic	Total	Total Sedentary Behavior Quartiles (hours/day)			
		Q1 (≤10.7)	Q2 (>10.7–11.8)	Q3 (>11.8–12.9)	Q4 (>12.9)
*Total N*	91,292	22,827	22,822	22,823	22,820
**Age, years, mean (SD)**	56.0 (7.8)	54.0 (7.8)	55.4 (7.8)	56.5 (7.7)	58.2 (7.4)
**Sex (%)**					
Female	51,169 (56.0)	14,251 (62.4)	13,294 (58.3)	12,585 (55.1)	11,039 (48.4)
Male	40,123 (44.0)	8,576 (37.6)	9,528 (41.7)	10,238 (44.9)	11,781 (51.6)
**Deprivation index, mean (SD)**	−1.7 (2.8)	−1.9 (2.7)	−1.8 (2.7)	−1.8 (2.8)	−1.4 (3.0)
**Ethnicity (%)**					
White	88,130 (96.9)	22,071 (97.0)	22,139 (97.3)	22,028 (96.9)	21,892 (96.4)
South Asian	861 (0.9)	184 (0.8)	194 (0.9)	220 (1.0)	263 (1.2)
Black	774 (0.9)	184 (0.8)	159 (0.7)	192 (0.8)	239 (1.1)
Chinese	209 (0.2)	63 (0.3)	43 (0.2)	53 (0.2)	50 (0.2)
Mixed	507 (0.6)	140 (0.6)	118 (0.5)	120 (0.5)	129 (0.6)
Other	493 (0.5)	116 (0.5)	108 (0.5)	131 (0.6)	138 (0.6)
**Education level (%)**					
College or University degree	39,375 (43.4)	9,247 (40.7)	9,889 (43.6)	10,292 (45.4)	9,947(43.9)
A levels/AS levels or equivalent	11,970 (13.2)	3,094 (13.6)	3,016 (13.3)	2,916 (12.9)	2,944 (13.0)
O levels/GCSEs or equivalent	18,505 (20.4)	4,907 (21.6)	4,703 (20.7)	4,492 (19.8)	4,403 (19.4)
SEs or equivalent	3,647 (4.0)	1,261 (5.6)	931 (4.1)	802 (3.5)	653 (2.9)
NVQ or HND or HNC or equivalent	4,873 (5.4)	1,263 (5.6)	1,182 (5.2)	1,179 (5.2)	1,249 (5.5)
Other professional qualifications	4,538 (5.0)	1,138 (5.0)	1,101 (4.9)	1,104 (4.9)	1,195 (5.3)
None of the above	7,453 (8.2)	1710 (7.5)	1,775 (7.8)	1,806 (8.0)	2,162 (9.5)
Prefer not to answer	382 (0.4)	96 (0.4)	86 (0.4)	87 (0.4)	113 (0.5)
**Never had sugary foods/drinks (%)**	13,287 (14.6)	3,091 (13.6)	3,179 (14.0)	3,300 (14.6)	3,717 (16.4)
**Dietary intake, mean (SD)**					
Fruits and vegetable intake, servings/day	4.2 (2.3)	4.3 (2.3)	4.2 (2.2)	4.2 (2.2)	4.1 (2.3)
Oil fish intake, times/week	1.1 (1.0)	1.1 (1.0)	1.1 (1.0)	1.1 (1.0)	1.1 (1.0)
Processed meat intake, times/week	1.4 (1.4)	1.3 (1.3)	1.4 (1.3)	1.4 (1.4)	1.5 (1.4)
Red meat intake, times/week	2.1 (1.4)	2.0 (1.4)	2.0 (1.4)	2.1 (1.4)	2.1 (1.4)
**Smoking, mean (SD)**					
Never	52,099 (57.2)	13,397 (58.9)	13,302 (58.4)	13,091 (57.5)	12,309 (54.1)
Previous	32,593 (35.8)	7,838 (34.4)	8,060 (35.4)	8,113 (35.6)	8,582 (37.7)
Current	6,354 (7.0)	1,527 (6.7)	1,411 (6.2)	1,554 (6.8)	1,862 (8.2)
**Alcohol consumption, units/week, mean (SD)**	15.9 (16.7)	15.9 (16.3)	16.0 (16.6)	15.7 (16.3)	15.9 (17.7)
**BMI, kg/m** ^**2**^ **, mean (SD)**	26.7 (4.5)	25.7 (4.1)	26.2 (4.2)	27.0 (4.4)	28.1 (4.9)
**WHR, mean (SD)**	0.86 (0.09)	0.84 (0.08)	0.85 (0.09)	0.86 (0.09)	0.88 (0.09)
**WHtR, mean (SD)**	0.52 (0.07)	0.50 (0.07)	0.51 (0.07)	0.52 (0.07)	0.55 (0.08)
**WC, cm, mean (SD)**	88.3 (13.1)	84.8 (12.2)	86.8 (12.4)	88.9 (12.7)	92.9 (13.6)
**Multimorbidity count, mean (SD)**	1.0 (1.1)	0.9 (1.0)	0.9 (1.1)	1.0 (1.1)	1.2 (1.2)
**Time use behaviors, hours/day, mean (SD)**					
Sleep	7.2 (1.4)	7.2 (1.6)	7.5 (1.3)	7.3 (1.2)	6.6 (1.3)
LPA	3.0 (1.0)	3.7 (1.2)	3.3 (0.8)	2.9 (0.7)	2.3 (0.7)
MPA	0.9 (0.6)	1.3 (0.8)	1.0 (0.5)	0.8 (0.4)	0.6 (0.4)
VPA	0.1 (0.1)	0.1 (0.1)	0.1 (0.1)	0.1 (0.1)	0.1 (0.1)
**Outcome (%)**					
Overall cancer mortality	1,726 (1.9)	300 (1.3)	358 (1.6)	431 (1.9)	637 (2.8)
Overall cancer incidence	12,392 (13.6)	2,542 (11.1)	2,916 (12.8)	3,192 (14.0)	3,742 (16.4)
Obesity-related cancer incidence	4,710 (5.2)	996 (4.4)	1,112 (4.9)	1,212 (5.3)	1,390 (6.1)
T2D-related cancer incidence	5,586 (6.1)	1,131 (5.0)	1,279 (5.6)	1,471 (6.4)	1,705 (7.5)

Abbreviations: Q1, first quartile; Q2, second quartile; Q3, third quartile; Q4, fourth quartile; LPA, light physical activity; MPA, moderate physical activity; VPA, vigorous physical activity; BMI, body mass index; WHtR, waist-to-height ratio; WHR, waist-to-hip ratio; WC, waist circumference.

Table 2 shows the associations between a 1-hour increment in overall, prolonged and interrupted SB and composite cancer outcomes. Each overall sedentary time was associated with a higher risk of overall cancer mortality (hazard ratio [HR]_1 hour_ 1.12; 95% confidence interval [CI] [1.08, 1.15]; *p* < 0.001); overall incident cancer (HR_1 hour_ 1.03; 95% CI [1.02, 1.04]; *p* < 0.001); obesity-related cancer (HR_1 hour_ 1.06; 95% CI [1.04, 1.08]; *p* < 0.001); and T2D-related cancer (HR_1 hour_ 1.07; 95% CI [1.05, 1.09]; *p* < 0.001). However, only prolonged SB was associated with a higher risks of cancer mortality (HR_1 hour_ 1.09; 95% CI [1.06, 1.11]; *p* < 0.001); overall incident cancer (HR_1 hour_ 1.03; 95% CI [1.02, 1.04]; *p* < 0.001); obesity-related cancer (HR_1 hour_ 1.05; 95% CI [1.03,1.06]; *p* < 0.001); and T2D-related cancer (HR_1 hour_ 1.05; 95% CI [1.04, 1.07]; *p* < 0.001). In contrast, interrupted SB was associated with a lower risk of all outcomes: cancer mortality (HR_1 hour_ 0.82; 95% CI [0.78, 0.86]; *p* < 0.001); overall incident cancer (HR_1 hour_ 0.94; 95% CI [0.92, 0.96]; *p* < 0.001); obesity-related cancer (HR_1 hour_ 0.91; 95% CI [0.88, 0.94]; *p* < 0.001); and T2D-related cancer (HR_1 hour_ 0.90; 95% CI [0.88, 0.93]; *p* < 0.001). Similar associations were observed for several site-specific cancers: breast, lung, and oral cancers, non-Hodgkin lymphoma and leukemia) (Table E in S1 Appendix). For example, overall and prolonged SB were associated with a higher risk of breast cancer, respectively (overall SB: HR_1 hour_ 1.05; 95% CI [1.02, 1.09]; *p* = 0.002; prolonged SB: HR_1 hour_ 1.04; 95% CI [1.02, 1.06]; *p* < 0.001), whereas interrupted SB was associated with a lower risk (HR_1 hour_ 0.92; 95% CI [0.88, 0.96]; *p* < 0.001). The associations between quartiles of total sedentary time (hours/day) and cancer outcomes are detailed in Table F in S1 Appendix.

**Table 2:** Association between 1 hour of sedentary behavior and composite cancer outcomes based on main model.

	Overall sedentary behavior		Prolonged sedentary behavior		Interrupted sedentary behavior	
	HR (95% CI)	*P*-value	HR (95% CI)	*P*-value	HR (95% CI)	*P*-value
**Model 1**						
**Overall cancer mortality**	1.10 (1.06, 1.14)	<0.001	1.10 (1.07, 1.12)	<0.001	0.81 (0.77, 0.85)	<0.001
**Overall cancer incidence**	1.03 (1.02, 1.04)	<0.001	1.03 (1.02, 1.04)	<0.001	0.93 (0.92, 0.95)	<0.001
**Obesity-related cancer**	1.07 (1.04, 1.09)	<0.001	1.05 (1.04, 1.07)	<0.001	0.90 (0.88, 0.93)	<0.001
**T2D-related cancer**	1.08 (1.06, 1.10)	<0.001	1.06 (1.05, 1.08)	<0.001	0.88 (0.86, 0.91)	<0.001
**Model 2**						
**Overall cancer mortality**	1.12 (1.08, 1.15)	<0.001	1.09 (1.06, 1.11)	<0.001	0.82 (0.78, 0.86)	<0.001
**Overall cancer incidence**	1.03 (1.02, 1.04)	<0.001	1.03 (1.02, 1.04)	<0.001	0.94 (0.92, 0.96)	<0.001
**Obesity-related cancer**	1.06 (1.04, 1.08)	<0.001	1.05 (1.03, 1.06)	<0.001	0.91 (0.88, 0.94)	<0.001
**T2D-related cancer**	1.07 (1.05, 1.09)	<0.001	1.05 (1.04, 1.07)	<0.001	0.90 (0.88, 0.93)	<0.001

Model 1 adjusted for age, sex, and ethnicity; Model 2 additionally adjusted for deprivation, education, smoking, intake of alcohol, sugar, processed meat, red meat, fruit and vegetables, and oil fish.

Obesity-related cancer including esophagus cancer, liver cancer, kidney cancer, myeloma, pancreatic cancer, colorectal cancer, gallbladder cancer, breast cancer, ovarian cancer, and thyroid cancer.

T2D-related cancers including thyroid cancer, breast cancer, liver cancer, pancreatic cancer, endometrial cancer, esophagus cancer, colorectal cancer, kidney cancer, gallbladder cancer, ovarian cancer, non-Hodgkin lymphoma, leukemia, and bladder cancer.

The two sensitivity analyses did not alter the findings materially. Additional adjustment for BMI and LTCs produced similar patterns for the associations between total, prolonged, and interrupted SB and composite cancer risk (Table G in S1 Appendix). After excluding the first two years of follow-up, overall and prolonged SB were still associated with increased cancer risk, while interrupted SB was still associated with reduced risk of all examined cancer outcomes (Table K in S1 Appendix).

**Fig 2:**
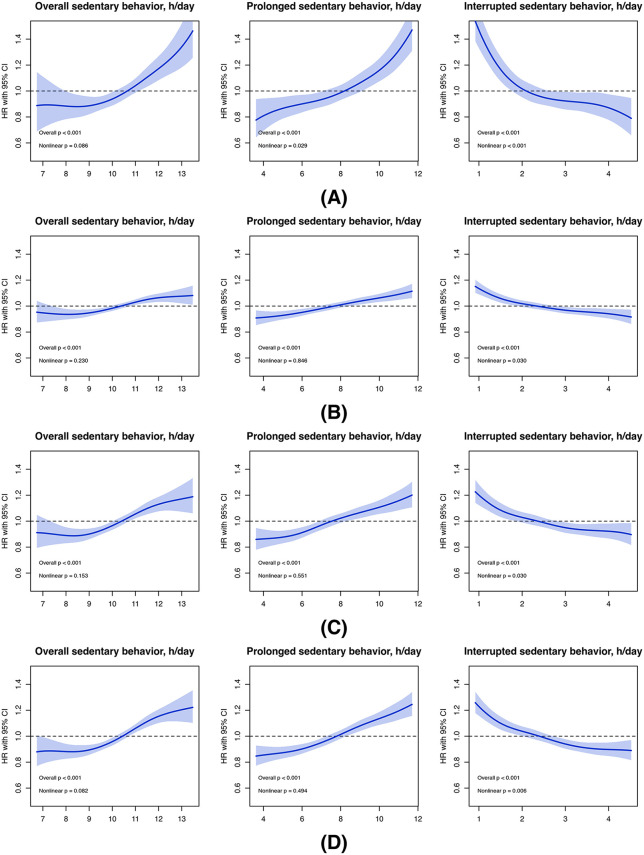
Nonlinear association between SB and composite cancer outcomes. All models were adjusted for age, sex, and ethnicity, deprivation, education, smoking, intake of alcohol, sugar, processed meat, red meat, fruit and vegetables, and oily fish. Obesity-related cancer including esophagus cancer, liver cancer, kidney cancer, myeloma, pancreatic cancer, colorectal cancer, gallbladder cancer, breast cancer, ovarian cancer, and thyroid cancer.T2D-related cancers including thyroid cancer, breast cancer, liver cancer, pancreatic cancer, endometrial cancer, esophagus cancer, colorectal cancer, kidney cancer, gallbladder cancer, ovarian cancer, non-Hodgkin lymphoma, leukemia, and bladder cancer. Panels **(A)**, **(B)**, **(C)**, and **(D)** represent the association between overall cancer mortality, the incidence of overall, obesity-related, and T2D-related cancers, respectively.

The nonlinear associations between SB and cancer mortality and T2D-related cancer were shown in Fig 2. The relationships between prolonged SB and cancer outcomes were approximately linear across the range of SB in the study cohort, whereas overall SB showed some evidence of nonlinearity. Interrupted SB was inversely associated with cancer outcomes, with the risk reduction being more pronounced at lower levels and then tending to level off at higher levels of interrupted SB. The nonlinear associations for PA are shown in Fig 1 in S1 Appendix.

The associations between different intensities of PA and composite cancer outcomes and site-specific cancers are shown in Tables H and I in S1 Appendix. VPA, even of relatively short duration, was strongly associated with lower risk of cancer mortality, T2D-related cancers, and cancers of esophagus, uterine, liver, colorectal, bladder, and lung, as well as leukemia.

Table 3 shows the interaction tests between SB and adiposity defined using BMI, WHR, and WHtR in relation to composite cancer outcomes. Overall, there was no significant interaction between total or prolonged sedentary time and general or central obesity for risks of overall cancer, obesity-related cancer, or T2D-related cancer.

**Table 3:** Interaction between sedentary behavior and adiposity indicator in composite cancer outcomes based on main model.

Outcome	Interaction with	Overall sedentary behavior		Prolonged sedentary behavior		Interrupted sedentary behavior	
		Ratio of HR (95% CI)	*P*-value	Ratio of HR (95% CI)	*P*-value	Ratio of HR (95% CI)	*P*-value
**Overall cancer mortality**	BMI ≥ 30 kg/m^2^	1.01 (0.93, 1.09)	0.846	1.00 (0.95, 1.05)	0.957	1.01 (0.90, 1.13)	0.889
**Overall cancer incidence**	BMI ≥ 30 kg/m^2^	1.00 (0.97, 1.02)	0.762	1.00 (0.98, 1.02)	0.743	1.01 (0.96, 1.05)	0.799
**Obesity-related cancer**	BMI ≥ 30 kg/m^2^	1.01 (0.96, 1.06)	0.756	1.01 (0.98, 1.04)	0.600	0.98 (0.91, 1.05)	0.508
**T2D-related cancer**	BMI ≥ 30 kg/m^2^	1.01 (0.97, 1.06)	0.569	1.00 (0.97, 1.03)	0.953	1.02 (0.96, 1.09)	0.475
**Overall cancer mortality**	WHtR ≥ 0.5	0.96 (0.89, 1.04)	0.352	0.98 (0.93, 1.03)	0.461	1.01 (0.91, 1.13)	0.831
**Overall cancer incidence**	WHtR ≥ 0.5	0.99 (0.96, 1.01)	0.337	0.99 (0.98, 1.01)	0.444	1.00 (0.97, 1.04)	0.837
**Obesity-related cancer**	WHtR ≥ 0.5	1.00 (0.96, 1.04)	0.956	1.00 (0.97, 1.03)	0.977	1.00 (0.94, 1.06)	0.871
**T2D-related cancer**	WHtR ≥ 0.5	1.02 (0.98, 1.06)	0.352	1.01 (0.99, 1.04)	0.333	0.98 (0.92, 1.03)	0.439
**Overall cancer mortality**	WHR ≥ 0.85 (F) and 0.90 (M)	1.00 (0.93, 1.06)	0.850	1.01 (0.96, 1.05)	0.755	0.95 (0.86, 1.05)	0.344
**Overall cancer incidence**	WHR ≥ 0.85 (F) and 0.90 (M)	0.99 (0.97, 1.02)	0.615	1.00 (0.98, 1.01)	0.682	1.00 (0.97, 1.04)	0.870
**Obesity-related cancer**	WHR ≥ 0.85 (F) and 0.90 (M)	1.01 (0.97, 1.05)	0.561	1.00 (0.98, 1.03)	0.726	1.00 (0.95, 1.06)	0.898
**T2D-related cancer**	WHR ≥ 0.85 (F) and 0.90 (M)	1.03 (0.99, 1.07)	0.167	1.01 (0.99, 1.04)	0.326	1.00 (0.95, 1.05)	0.943
**Overall cancer mortality**	WC ≥ 88 (F) and 102 (M) cm	0.98 (0.92, 1.05)	0.594	0.99 (0.94, 1.03)	0.544	1.03 (0.93, 1.14)	0.606
**Overall cancer incidence**	WC ≥ 88 (F) and 102 (M) cm	1.01 (0.96, 1.02)	0.472	0.99 (0.97, 1.01)	0.429	1.01 (0.97, 1.05)	0.511
**Obesity-related cancer**	WC ≥ 88 (F) and 102 (M) cm	1.01 (0.97, 1.05)	0.715	1.01 (0.98, 1.04)	0.391	0.96 (0.90, 1.02)	0.189
**T2D-related cancer**	WC ≥ 88 (F) and 102 (M) cm	1.01 (0.97, 1.05)	0.609	1.01 (0.98, 1.03)	0.669	0.99 (0.94, 1.05)	0.855

Ratio of HR is the multiplicative interaction in the Cox regression model, representing HR in adiposity group/ HR in nonadiposity group. For example, the 1.01 of high BMI and overall sedentary behavior on overall cancer mortality indicates that people with higher BMI had 1% higher HR than those with lower BMI.

Model adjusted for age, sex, ethnicity, deprivation, education, smoking, intake of alcohol, sugar, intake of alcohol, sugar, processed meat, red meat, fruit and vegetables, and oil fish.

Obesity-related cancer including esophagus cancer, liver cancer, kidney cancer, myeloma, pancreatic cancer, colorectal cancer, gallbladder cancer, breast cancer, ovarian cancer, and thyroid cancer.

T2D-related cancers including thyroid cancer, breast cancer, liver cancer, pancreatic cancer, endometrial cancer, esophagus cancer, colorectal cancer, kidney cancer, gallbladder cancer, ovarian cancer, non-Hodgkin lymphoma, leukemia, and bladder cancer.

Abbreviations: BMI, body mass index; WHtR, waist-to-height ratio; WHR, waist-to-hip ratio; WC, waist circumference.

Table 4 presents hazard ratios for composite cancer outcomes associated with specified reallocation of time from one type of sedentary behavior to another behavior. Replacing 1 hour per day of prolonged SB with other behaviors was generally associated with lower risks of the composite cancer outcomes. For example, replacing 1 hour of prolonged SB with LPA was associated with 12% lower hazard (HR 0.88; 95% CI [0.79, 0.99]; *p* = 0.033). Replacing 5 min/day of prolonged SB with 5 min/day VPA was associated with the largest reduction in cancer incidence outcomes (HR_T2D-related cancer_ 0.89; 95% CI [0.86, 0.92; *p* < 0.001]). Hazard ratios for site-specific cancer outcomes under the same reallocation scenarios are presented in Table J in S1 Appendix.

**Table 4:** Hazard ratios for composite cancer outcomes associated with replacing type-specific SB with other SB and physical activity in isotemporal substitution models.

	Overall cancer mortality		Overall cancer incidence		Obesity-related cancer		T2D-related cancer	
	HR (95% CI)	P-value	HR (95% CI)	P-value	HR (95% CI)	P-value	HR (95% CI)	P-value
**Replacing prolonged SB with:**								
Interrupted SB (1h/d)	0.97 (0.87, 1.08)	0.600	0.99 (0.96, 1.03)	0.700	1.01 (0.95, 1.08)	0.700	1.02 (0.96, 1.08)	0.600
LPA (1h/d)	0.88 (0.79, 0.99)	0.033	0.94 (0.90, 0.98)	0.003	0.90 (0.84, 0.96)	0.001	0.89 (0.83, 0.94)	<0.001
MPA (30mins/d)	0.92 (0.86, 0.99)	0.024	0.99 (0.96, 1.01)	0.300	0.99 (0.95, 1.03)	0.500	0.97 (0.94, 1.01)	0.120
VPA (5mins/d)	0.78 (0.46, 1.30)	0.300	0.96 (0.94, 0.98)	<0.001	0.91 (0.88, 0.95)	<0.001	0.89 (0.89, 0.94)	<0.001
**Replacing interrupted SB with:**								
Prolonged SB (1h/d)	1.01 (0.97, 1.05)	0.700	1.01 (0.99, 1.02)	0.400	1.02 (1.00, 1.04)	0.100	1.02 (1.00, 1.04)	0.061
LPA (1h/d)	0.87 (0.80, 0.95)	0.001	0.95 (0.92, 0.98)	<0.001	0.93 (0.89, 0.98)	0.008	0.93 (0.89, 0.97)	0.003
MPA (30mins/d)	0.92 (0.86, 0.98)	0.009	0.99 (0.97, 1.01)	0.300	1.01 (0.97, 1.04)	0.700	0.99 (0.96, 1.03)	0.700
VPA (5mins/d)	0.77 (0.46, 1.29)	0.300	0.96 (0.94, 0.98)	<0.001	0.91 (0.88, 0.95)	<0.001	0.89 (0.86, 0.92)	<0.001

## Discussion

Overall time spent in SB is associated with adverse health outcomes. However, in this large population cohort, we demonstrated substantial differences between prolonged and interrupted SB. More time spent in prolonged SB was associated with higher risk of a wide range of cancer outcomes, whilst more time spent in interrupted SB was associated with a lower risk. Replacing prolonged SB with additional PA was associated with additional reductions in risk, even if it was replaced by light intensity PA. To our knowledge, this is the first study that showed the differential associations of interrupted and prolonged SB with cancer outcomes, using state-of-the-art classification methods and objectively measured time use activities.

Our findings are generally consistent with, but meaningfully extend, existing studies that utilized accelerometer-measured data. For instance, a prospective study [34] involving 8,002 US middle-aged and older participants found that both total sedentary time and sedentary bout duration were associated with significantly higher cancer mortality risks. In the unadjusted model, these sedentary behaviors were associated with 82% (95% CI [1.27, 2.60]; *p* < 0.001) and 61% (95% CI [1.16, 2.24]; *p* = 0.005) higher risk of cancer mortality, respectively. Another prospective study [35] involving 7,671 European participants, among whom 516 cancer events were recorded, found that SB accumulated in longer bout durations was associated with higher rates of incident cancer. Specifically, prolonged sedentary bouts were associated with an increased risk of cancer incidence 12% (95% CI [1.02, 1.23]; *p* = 0.023), which is consistent with the association between prolonged SB and overall cancer incidence observed in our study. Beyond studies examining composite cancer outcomes and those using accelerometer-measured data, one observational study [36] investigated the associations between overall SB and the risks of colon, endometrial, and lung cancers. Total SB was associated with 24%, 32%, and 21% increased risk for these cancers, respectively, which is also broadly consistent with some of our site-specific findings.

The ISM results from this study are also consistent with the accelerometer-based findings reported by Gilchrist and colleagues [34], who found that replacing 30 min of total sedentary time with either LPA or moderate-to-vigorous PA (MVPA) was associated with a lower risk of cancer mortality. Specifically, a 30-min replacing was associated with an 8% reduction for LPA (HR 0.92, 95% CI [0.86, 0.97]) and a 31% reduction for MVPA (HR 0.69, 95% CI [0.48, 0.97]). Additionally, that study reported linear dose-response associations of both total sedentary time and mean sedentary bout length with cancer mortality. Total sedentary time was significantly associated with cancer mortality risk in a linear, dose-response (HR per 1 hour/d increase in total sedentary time 1.16, 95% CI [1.03, 1.31]). However, this study focused solely on the association between SB and cancer mortality, without addressing cancer incidence. They also did not explicitly examine differences between interrupted and prolonged SB.

Our findings suggest that the health effects of SB may depend not only on total sedentary time, but also on whether that time is accumulated in prolonged bouts or interrupted by activity. This pattern is biologically plausible: experimental studies have shown that interrupting prolonged sitting with short bouts of activity can improve metabolic responses compared with uninterrupted sitting [37,38]. Prolonged sedentary behaviors were found to promote chronic low-grade inflammation [39] as well as suppressing efficient immune function [40]. In particular, higher device-measured total SB has been associated with endothelial dysfunction and low-grade inflammation independent of PA levels [41]. However, that study found no significant associations for sedentary breaks or average sedentary bout length, possibly because sedentary interruption was operationalized differently and the sample size was smaller (*n* = 2,363).

Additional evidence suggests that replacing SB with PA may contribute to cancer prevention through several biological pathways that are not limited to body weight [25]. One possible pathway is ectopic fat accumulation [42] whereby triglyceride are deposited in nonadipose tissues such as the liver, skeletal muscles, heart, and pancreas. This pattern of fat storage was found to be particularly prevalent in people who were sedentary [43] and is metabolically harmful, explaining the links between sedentary behavior and cancer risk [25,44]. Prolonged sedentary time may also contribute to impaired glucose regulation and hyperinsulinemia. Insulin and insulin-like growth factor I (IGF-I) regulate glucose metabolism, cell growth, apoptosis, and angiogenesis, and chronic upregulation of these pathways has been linked to several cancers, including breast, prostate, and colorectal cancer [36]. The precise molecular mechanisms underlying these associations could not be examined in this study and warrant future investigation.

Our study has several strengths. First and foremost, it used device-measured PA, which should accurately reflect the actual SB and PA level of participants and is robust against recall or reporting bias. Second, we used a validated, machine learning-informed approach to derive SB, which is likely to be more accurate than questionnaire-based assessment and more informative than traditional cut-point methods [45]. Finally, to our knowledge, our study is the first comprehensive study that explored the associations of type-specific SB with both composite and individual cancer outcomes. However, our study is subject to the following limitations. Firstly, device-measured PA only captured behaviors in a limited time window, which may not necessarily represent the long-term habitual behavior. Importantly, even though we could characterize SB by their bout length, there may be other contextual factors related to PA that could modulate the association. For example, a previous UK Biobank study has found self-reported television viewing, but not driving or using a computer, to be associated with higher cancer risk [46]. Currently, there are no reliable methods to map objectively measured SB to its context and would warrant future studies. Secondly, UK Biobank is not representative of the UK population, with evidence of healthy volunteer bias. Estimates of effect size were found to be consistent with population-representative cohorts, but PA levels are higher among UK Biobank participants. Thirdly, despite best efforts, there are limitations on how cancer outcomes were defined. For example, the number of events was not sufficient to separate leukemia by cell lineage, and there was no information on the proportion of esophageal adenocarcinoma in esophageal cancers. Last but not least, as with other observational studies, residual and occupation confounding cannot be ruled out and could be, partly, driving the observed associations. Specifically, general frailty or LTCs that could affect mobility could be major confounders, which have been mitigated in this study via a long-term condition variable. However, it should also be worth noting SB could also affect the development of LTCs and the adjustment of that variable could induce overadjustment bias.

The findings of this study showed that replacing prolonged SB as well as replacing SB with PA may play a role in cancer prevention. Individuals who accumulate large amounts of prolonged sedentary behavior may therefore be an important target group for future interventions. There are multiple ways to intervene, including breaking down of prolonged sedentary behavior, and replacing that by PA of different intensity. If a person could replace SB with PA, the potential cancer risk reduction is dependent on the PA intensity. Not surprisingly, replacing even 5 min a day of prolonged sedentary time with VPA is associated with the lowest cancer incidence risk [20], which has also been reported previously in analyses of the same UK Biobank accelerometer dataset using a different exposure definition [20]. However, it should be noted that VPA may not be feasible for most people, especially for those who currently spend substantial time in SB—they are most likely to be older, frail, and have multimorbidity [47]. For these individuals, replacing SB with LPA might be a more achievable target, and the study showed that LPA is already associated with tangibly lower risk. It should be noted that LPA is currently not included in any of the PA guidelines, partly because of the lack of measurement—it is hard to measure LPA using questionnaires—and this study showed that LPA should not be overlooked. At the same time, several hazard ratios were modest in magnitude and should not be overinterpreted at the individual clinical level. However, some replacement could still be potentially meaningful, e.g., replacing prolonged sedentary time with LPA was associated with approximately a sizable reduction of cancer mortality and obesity- and T2D-related cancer incidence. Given the high prevalence of sedentary behavior, even modest associations could still have important public health implications, although formal cost-effectiveness analyses would be needed before recommending resource-intensive interventions.

In summary, prolonged, but not interrupted, SB was associated with higher risks of a wide range of cancer outcomes. Replacing prolonged SB with PA was associated with lower risks of cancer outcomes. These findings should be corroborated in intervention studies.

## Supporting information

S1 Appendix Supplementary Tables A–K and Fig A. **Table A.** Long-term morbidity groupings. This table lists the long-term morbidity categories used in the analysis and their corresponding definitions or codes. ICD-10, International Classification of Diseases, 10th Revision. **Table B.** Site specific cancers incidence of participants by total sedentary behavior quartiles. Values are presented as numbers (%) unless otherwise stated. Q1, first quartile; Q2, second quartile; Q3, third quartile; Q4, fourth quartile. **Table C.** Baseline characteristics of participants by prolonged sedentary behavior quartiles. Values are presented as number (%) unless otherwise stated.: Q1, first quartile; Q2, second quartile; Q3, third quartile; Q4, fourth quartile; LPA, light physical activity; MPA, moderate physical activity; VPA, vigorous physical activity; BMI, body mass index; WHtR, waist-to-height ratio; WHR, waist-to-hip ratio; WC, waist circumference. **Table D.** Baseline characteristics of participants by interrupted sedentary behavior quartiles. Values are presented as number (%) unless otherwise stated. Q1, first quartile; Q2, second quartile; Q3, third quartile; Q4, fourth quartile; LPA, light physical activity; MPA, moderate physical activity; VPA, vigorous physical activity; BMI, body mass index; WHtR, waist-to-height ratio; WHR, waist-to-hip ratio; WC, waist circumference. **Table E.** Association between sedentary behavior and site-specific cancers. All models were adjusted for age, sex, and ethnicity, deprivation, education, smoking, intake of alcohol, sugar, processed meat, red meat, fruit and vegetables, and oily fish. **Table F.** Association between total sedentary behavior and composite cancer outcomes. Q1, first quartile; Q2, second quartile; Q3, third quartile; Q4, fourth quartile. All models were adjusted for age, sex, and ethnicity, deprivation, education, smoking, intake of alcohol, sugar, processed meat, red meat, fruit and vegetables, and oily fish. Obesity-related cancer including esophagus cancer, liver cancer, kidney cancer, myeloma, pancreatic cancer, colorectal cancer, gallbladder cancer, breast cancer, ovarian cancer, and thyroid cancer.T2D-related cancers including thyroid cancer, breast cancer, liver cancer, pancreatic cancer, endometrial cancer, esophagus cancer, colorectal cancer, kidney cancer, gallbladder cancer, ovarian cancer, non-Hodgkin lymphoma, leukemia, and bladder cancer. **Table G.** Sensitivity analysis of the association between sedentary behavior and composite cancer risk adjusted for BMI and morbidity count. All models were adjusted for age, sex, and ethnicity, deprivation, education, smoking, intake of alcohol, sugar, processed meat, red meat, fruit and vegetables, and oily fish. Obesity-related cancer including esophagus cancer, liver cancer, kidney cancer, myeloma, pancreatic cancer, colorectal cancer, gallbladder cancer, breast cancer, ovarian cancer, and thyroid cancer.T2D-related cancers including thyroid cancer, breast cancer, liver cancer, pancreatic cancer, endometrial cancer, esophagus cancer, colorectal cancer, kidney cancer, gallbladder cancer, ovarian cancer, non-Hodgkin lymphoma, leukemia, and bladder cancer. **Table H.** Association between intensity of physical activity and composite cancer outcomes. All models were adjusted for age, sex, and ethnicity, deprivation, education, smoking, intake of alcohol, sugar, processed meat, red meat, fruit and vegetables, and oily fish. Obesity-related cancer including esophagus cancer, liver cancer, kidney cancer, myeloma, pancreatic cancer, colorectal cancer, gallbladder cancer, breast cancer, ovarian cancer, and thyroid cancer.T2D-related cancers including thyroid cancer, breast cancer, liver cancer, pancreatic cancer, endometrial cancer, esophagus cancer, colorectal cancer, kidney cancer, gallbladder cancer, ovarian cancer, non-Hodgkin lymphoma, leukemia, and bladder cancer. **Table I.** Association between intensity of physical activity and site-specific cancers. All models were adjusted for age, sex, and ethnicity, deprivation, education, smoking, intake of alcohol, sugar, processed meat, red meat, fruit and vegetables, and oily fish. LPA, light physical activity; MPA, moderate physical activity; VPA, vigorous physical activity. **Table J.** Hazard ratios for site specific cancer outcomes associated with replacing type-specific SB with other SB and physical activity in isotemporal substitution models. All models were adjusted for age, sex, and ethnicity, deprivation, education, smoking, intake of alcohol, sugar, processed meat, red meat, fruit and vegetables, and oily fish. LPA, light physical activity; MPA, moderate physical activity; VPA, vigorous physical activity; ISB, interrupted sedentary behavior. **Table K.** Association between sedentary behavior and incident of composite cancer, excluding first 2 years of follow-up. All models were adjusted for age, sex, and ethnicity, deprivation, education, smoking, intake of alcohol, sugar, processed meat, red meat, fruit and vegetables, and oily fish. Obesity-related cancer including esophagus cancer, liver cancer, kidney cancer, myeloma, pancreatic cancer, colorectal cancer, gallbladder cancer, breast cancer, ovarian cancer, and thyroid cancer.T2D-related cancers including thyroid cancer, breast cancer, liver cancer, pancreatic cancer, endometrial cancer, esophagus cancer, colorectal cancer, kidney cancer, gallbladder cancer, ovarian cancer, non-Hodgkin lymphoma, leukemia, and bladder cancer. **Fig A.** Nonlinear association between intensity of physical activity and composite cancer outcomes. All models were adjusted for age, sex, and ethnicity, deprivation, education, smoking, intake of alcohol, sugar, processed meat, red meat, fruit and vegetables, and oily fish. Obesity-related cancer including esophagus cancer, liver cancer, kidney cancer, myeloma, pancreatic cancer, colorectal cancer, gallbladder cancer, breast cancer, ovarian cancer, and thyroid cancer. T2D-related cancers including thyroid cancer, breast cancer, liver cancer, pancreatic cancer, endometrial cancer, esophagus cancer, colorectal cancer, kidney cancer, gallbladder cancer, ovarian cancer, non-Hodgkin lymphoma, leukemia, and bladder cancer. Panels (A), (B), (C), and (D) represent the association between overall cancer mortality, the incidence of overall, obesity-related, and T2D-related cancers, respectively.
(DOCX)

S1 Checklist STROBE Statement. The checklist of items that should be included in reports of cohort studies. The STROBE checklist is best used in conjunction with this article (freely available on the websites of PLoS Medicine at http://www.plosmedicine.org/, Annals of Internal Medicine at http://www.annals.org/, and Epidemiology at http://www.epidem.com/). Information on the STROBE Initiative is available at http://www.strobe-statement.org.
(DOCX)

## Abbreviations

BMI: body mass index
CI: confidence interval
CVD: cardiovascular disease
HR: hazard ratio
ICD-10: International Classification of Diseases, 10th revision
IGF-I: insulin-like growth factor I
ISM: Isotemporal substitution models
LTCs: long-term conditions
METs: metabolic equivalents of task
MLTCs: multiple long-term conditions
MVPA: moderate-to-vigorous PA
PA: physical activity
RCT: randomized controlled trial
SB: sedentary behavior
SBRN: Sedentary Behavior Research Network
SDs: standard deviations
T2D: type-2 diabetes
WC: waist circumference
WHR: waist-to-hip ratio
WHtR: waist-to-height ratio
