# Supplementary material for: Accelerometry-measured prolonged and interrupted sedentary behavior and cancer incidence and mortality: A cohort study of 91,292 UK Biobank participants
Source: PLoS Med. 2026 Jul 2;23(7):e1004767. doi: 10.1371/journal.pmed.1004767 (PMC13327276; doi:10.1371/journal.pmed.1004767)
Supplement: S1 Appendix — Table A. Long-term morbidity groupings. This table lists the long-term morbidity categories used in the analysis and their corresponding definitions or codes. ICD-10, International Classification of Diseases, 10th Revision. Table B. Site specific cancers incidence of participants by total sedentary behavior quartiles. Values are presented as numbers (%) unless otherwise stated. Q1, first quartile; Q2, second quartile; Q3, third quartile; Q4, fourth quartile. Table C. Baseline characteristics of participants by prolonged sedentary behavior quartiles. Values are presented as number (%) unless otherwise stated.: Q1, first quartile; Q2, second quartile; Q3, third quartile; Q4, fourth quartile; LPA, light physical activity; MPA, moderate physical activity; VPA, vigorous physical activity; BMI, body mass index; WHtR, waist-to-height ratio; WHR, waist-to-hip ratio; WC, waist circumference. Table D. Baseline characteristics of participants by interrupted sedentary behavior quartiles. Values are presented as number (%) unless otherwise stated. Q1, first quartile; Q2, second quartile; Q3, third quartile; Q4, fourth quartile; LPA, light physical activity; MPA, moderate physical activity; VPA, vigorous physical activity; BMI, body mass index; WHtR, waist-to-height ratio; WHR, waist-to-hip ratio; WC, waist circumference. Table E. Association between sedentary behavior and site-specific cancers. All models were adjusted for age, sex, and ethnicity, deprivation, education, smoking, intake of alcohol, sugar, processed meat, red meat, fruit and vegetables, and oily fish. Table F. Association between total sedentary behavior and composite cancer outcomes. Q1, first quartile; Q2, second quartile; Q3, third quartile; Q4, fourth quartile. All models were adjusted for age, sex, and ethnicity, deprivation, education, smoking, intake of alcohol, sugar, processed meat, red meat, fruit and vegetables, and oily fish. Obesity-related cancer including esophagus cancer, liver cancer, kidney canc [file pmed.1004767.s001.docx]

**S1 Appendix for**

**Accelerometry-measured prolonged and interrupted sedentary behavior and cancer incidence and mortality: A cohort study of 91,292 UK Biobank participants**

Ziyi Zhou (MPH) ^1 2 3^, Stewart G. Trost (PhD) ^4^, Gemma C. Ryde (PhD) ^5^,Solange Parra-Soto (PhD) ^6^, Zhe Fang (MBBS, MS) ^2^,Chao Xu (MSc) ^1^, Yujia Lu (MS) ^2^, Kai Wang (PhD) ^2^, Mengxi Du (PhD) ^2^ , Zhi Li (PhD) ^7^, Yuebin Lv (PhD) ^6^, Jason M.R. Gill (PhD) ^5^, Stuart R Gray (PhD) ^5^, Carlos Celis-Morales (PhD) ^5 8 9^, Marc J Gunter ^10 11^(PhD), Edward Giovannucci (MD, ScD)^12^, Jill P Pell (MD) ^1^, Mingyang Song (ScD) ^2 3 12 13 14 &^, Frederick K Ho (PhD) ^1^*^&^

**^&^Joint senior authors**

1. School of Health and Wellbeing, University of Glasgow, Glasgow, UK.
2. Department of Epidemiology, Harvard T.H. Chan School of Public Health, Boston, Massachusetts, United States of America.
3. Clinical and Translational Epidemiology Unit, Massachusetts General Hospital and Harvard Medical School, Boston, Massachusetts, United States of America.
4. School of Human Movement and Nutrition Sciences, The University of Queensland, Brisbane, Australia.
5. School of Cardiovascular and Metabolic Health, University of Glasgow, Glasgow, UK.
6. Department of Nutrition and Public Health, Universidad del Bío-Bío, Chillan, Chile.
7. China CDC Key Laboratory of Environment and Population Health, National Institute of Environmental Health, Chinese Center for Disease Control and Prevention, Beijing, China.
8. Human Performance Laboratory, Education, Physical Activity and Health Research Unit, Universidad Catolica del Maule, Talca, Chile.
9. High-Altitude Medicine Research Centre (CEIMA), Universidad Arturo Prat, Iquique, Chile.
10. Nutrition and Metabolism Branch, International Agency for Research on Cancer, World Health Organization, Lyon, France.
11. Department of Epidemiology and Biostatistics, School of Public Health, Imperial College London, London, UK.
12. Department of Nutrition, Harvard T.H. Chan School of Public Health, Boston, Massachusetts, United States of America.
13. Division of Gastroenterology, Massachusetts General Hospital and Harvard Medical School, Boston, Massachusetts, United States of America.
14. Broad Institute of MIT and Harvard, Cambridge, Massachusetts, United States of America.

^*^frederick.ho@glasgow.ac.uk

Table of Contents

[Table A. Long-term morbidity groupings. 3](#_Toc231301808)

[Table B. Site specific cancers incidence of participants by total sedentary behavior quartiles. 10](#_Toc231301809)

[Table C. Baseline characteristics of participants by prolonged sedentary behavior quartiles. 11](#_Toc231301810)

[Table D. Baseline characteristics of participants by interrupted sedentary behavior quartiles. 13](#_Toc231301811)

[Table E. Association between sedentary behavior and site-specific cancers. 15](#_Toc231301812)

[Table F. Association between total sedentary behavior and composite cancer outcomes. 17](#_Toc231301813)

[Table G. Sensitivity analysis of the association between sedentary behavior and composite cancer risk adjusted for BMI and morbidity count. 18](#_Toc231301814)

[Table I. Association between intensity of physical activity and site-specific cancers. 20](#_Toc231301815)

[Table J. Hazard ratios for site specific cancer outcomes associated with replacing type-specific SB with other SB and physical activity in isotemporal substitution models. 21](#_Toc231301816)

[Table K. Association between sedentary behavior and incident of composite cancer, excluding first two years of follow-up. 24](#_Toc231301817)

[Fig A. Non-linear association between intensity of physical activity and composite cancer outcomes. 25](#_Toc231301818)

# Table A. Long-term morbidity groupings.

| Morbidity grouping | Conditions included |
| --- | --- |
| 1. Painful conditions* | Back pain  Joint pain  Headaches (not migraine)  Sciatica Plantar fasciitis  Carpal tunnel syndrome  Fibromyalgia  Arthritis  Shingles  Disc problem  Prolapsed disc/slipped disc  Spine arthritis/spondylitis  Ankylosing spondylitis  Back problem  Osteoarthritis  Gout  Cervical spondylosis  Trigeminal neuralgia  Disc degeneration  Trapped nerve/compressed nerve |
| 2. Hypertension | Hypertension  Essential hypertension |
| 3. Depression* | Depression  Postnatal depression |
| 4. Asthma | Asthma |
| 5. Coronary Heart Disease | Heart attack/MI  Angina |
| 6. Treated dyspepsia | Gastro-oesophageal reflux (GORD)/gastric reflux  Esophagitis /Barrett's oesophagus  Gastric stomach ulcers  Gastric erosions/gastritis  Duodenal ulcer Dyspepsia/indigestion  Hiatus hernia  Helicobacter pylori |
| 7. Diabetes | Diabetic nephropathy  Diabetic neuropathy/ulcers  Diabetes Type 1 diabetes  Type 2 diabetes  Diabetic eye disease |
| 8. Thyroid disorders | Thyroid problem (not cancer)  Hyperthyroidism/thyrotoxicosis  Hypothyroidism/myxoedema  Graves’ disease  Thyroid goitre  Thyroiditis |
| 9. Rheumatoid arthritis, other inflammatory polyarthropathies, systemic connective tissue disorders and systemic autoimmune disorders | Myositis/myopathy  Systemic Lupus Erythematosus  Connective tissue disorder  Sjogren’s syndrome/sicca syndrome  Dermatopolymyositis  Scleroderma/systemic sclerosis  Rheumatoid arthritis  Psoriatic arthropathy  Dermatomyositis  Polymyositis  Polymyalgia Rheumatica  Malabsorption/coeliac disease |
| 10. Chronic Obstructive Pulmonary Disease (COPD) | COPD/chronic obstructive airways disease  Emphysema/chronic bronchitis  Emphysema |
| 11. Anxiety, other neurotic, stress related and somatoform disorders* | Anxiety/panic attacks  Nervous breakdown  Post-traumatic stress disorder  Obsessive compulsive disorder  Stress  Insomnia  Psychological/psychiatric problem |
| 12. Irritable bowel syndrome | Irritable bowel syndrome |
| 13. Alcohol problems* | Alcohol dependency  Alcoholic liver disease/alcoholic cirrhosis |
| 14. Other psychoactive substance abuse* | Opioid dependency  Other substance abuse/dependency |
| 15. Treated constipation | Constipation |
| 16. Stroke and Transient Ischaemic Attack (TIA) | Stroke TIA  Subarachnoid haemorrhage  Brain haemorrhage  Ischaemic stroke |
| 17. Chronic kidney disease | Polycystic kidney  Diabetic nephropathy  Renal/kidney failure  Renal failure requiring dialysis  Renal failure not requiring dialysis  Kidney nephropathy  Immunoglobulin A (IgA) nephropathy |
| 18. Diverticular disease of intestine | Diverticular disease/diverticulitis |
| 19. Atrial fibrillation | Atrial fibrillation |
| 20. Peripheral vascular disease | Peripheral vascular disease  Leg claudication/intermittent claudication |
| 21. Heart failure | Cardiomyopathy  Hypertrophic cardiomyopathy  Heart failure/pulmonary oedema |
| 22. Prostate disorders | Prostate problem (not cancer)  Enlarged prostate  Benign prostatic hypertrophy |
| 23. Glaucoma | Glaucoma |
| 24. Epilepsy | Epilepsy |
| 25. Dementia | Dementia/Alzheimer/cognitive impairment |
| 26. Schizophrenia (and related non-organic psychosis) and bipolar disorder* | Schizophrenia  Mania/bipolar disorder/manic depression |
| 27. Psoriasis or eczema | Eczema/dermatitis  Psoriasis |
| 28. Inflammatory bowel disease | Inflammatory bowel disease  Crohn’s disease  Ulcerative colitis |
| 29. Migraine | Migraine |
| 30. Chronic sinusitis | Chronic sinusitis |
| 31. Anorexia or bulimia* | Anorexia, bulimia/other eating disorder |
| 32. Bronchiectasis | Bronchiectasis |
| 33. Parkinson's disease | Parkinson's disease |
| 34. Multiple sclerosis | Multiple sclerosis |
| 35. Viral Hepatitis | Infective/viral hepatitis  Hepatitis B  Hepatitis C  Hepatitis D  Hepatitis E |
| 36. Chronic liver disease | Oesophageal varices  Non infective hepatitis  Liver failure/cirrhosis  Primary biliary cirrhosis |
| 37. Osteoporosis~ | Osteoporosis |
| 38. Chronic fatigue syndrome~ | Chronic fatigue syndrome |
| 39. Endometriosis~ | Endometriosis |
| 40. Meniere disease~ | Meniere disease |
| 41. Pernicious Anaemia~ | Pernicious anaemia |
| 42. Polycystic ovaries~ | Polycystic ovaries |
| 43. Cancers | Lifetime diagnosis  Female cancers  Male cancers |
| ^ Self-report lifetime diagnosis by doctor recorded by nurse-led interview (UK Biobank data field 20002), except cancer diagnosis which was reported by touch-screen questionnaire (UK Biobank data field 2453). The list of disease groupings was based on Barnett K, Mercer SW, Norbury M, Watt G, Wyke S, Guthrie B: Epidemiology of multimorbidity and implications for health care, research, and medical education: a cross-sectional study. Lancet 2012, 380(9836):37–43.(44)  ~Plus other conditions considered as long-term, requiring medication and that had a prevalence of ≥0. 1% in the whole UK Biobank cohort. | |

# Table B. Site specific cancers incidence of participants by total sedentary behavior quartiles.

| **Characteristic** | **Total** | **Total Sedentary Behavior Quartiles** | | | |
| --- | --- | --- | --- | --- | --- |
|  |  | **Q1 (≤10.7)** | **Q2 (>10.7 – 11.8)** | **Q3 (>11.8 – 12.9)** | **Q4 (>12.9)** |
| *Total N* | 91,292 | 22,827 | 22,822 | 22,823 | 22,820 |
| **Overall cancer mortality** | 1,726 (1.9) | 300 (1.3) | 358 (1.6) | 431 (1.9) | 637 (2.8) |
| **Overall cancer incidence** | 12,392 (13.6) | 2,542 (11.1) | 2,916 (12.8) | 3,192 (14.0) | 3,742 (16.4) |
| **Obesity-related cancer incidence** | 4,710 (5.2) | 996 (4.4) | 1,112 (4.9) | 1,212 (5.3) | 1,390 (6.1) |
| **T2D-related cancer incidence** | 5586 (6.1) | 1,131 (5.0) | 1,279 (5.6) | 1,471 (6.4) | 1,705 (7.5) |
| **Esophagus Cancer** | 196 (0.2) | 28 (0.1) | 42 (0.1) | 55 (0.2) | 71 (0.3) |
| **Kidney Cancer** | 265 (0.3) | 42 (0.2) | 56 (0.2) | 71 (0.3) | 96 (0.4) |
| **Uterine Cancer** | 303 (0.3) | 56 (0.2) | 71 (0.3) | 87 (0.4) | 89 (0.4) |
| **Liver Cancer** | 126 (0.1) | 20 (0.1) | 23 (0.1) | 34 (0.1) | 49 (0.2) |
| **Breast Caner** | 2,049 (2.2) | 481 (2.1) | 521 (2.3) | 540 (2.4) | 507 (2.2) |
| **Pancreas Cancer** | 217 (0.2) | 37 (0.2) | 42 (0.2) | 51 (0.2) | 87 (0.4) |
| **Colorectal Cancer** | 1,129 (1.2) | 234 (1.0) | 234 (1.0) | 291 (1.3) | 370 (1.6) |
| **Bladder Cancer** | 521 (0.6) | 89 (0.4) | 125 (0.5) | 135 (0.6) | 172 (0.8) |
| **Oral Cancer** | 201 (0.2) | 37 (0.2) | 54 (0.2) | 41 (0.2) | 69 (0.3) |
| **Lung Cancer** | 469 (0.5) | 84 (0.4) | 91 (0.4) | 109 (0.5) | 185 (0.8) |
| **Melanoma Cancer** | 575 (0.6) | 117 (0.5) | 159 (0.7) | 125 (0.5) | 174 (0.8) |
| **Ovary Cancer** | 204 (0.2) | 46 (0.2) | 52 (0.2) | 50 (0.2) | 56 (0.2) |
| **Prostate Cancer** | 2,231 (2.4) | 413 (1.8) | 508 (2.2) | 572 (2.5) | 738 (3.2) |
| **Non-Hodgkin lymphoma** | 480 (0.5) | 82 (0.4) | 104 (0.5) | 132 (0.6) | 162 (0.7) |
| **Multiple myeloma** | 180 (0.2) | 28 (0.1) | 38 (0.2) | 46 (0.2) | 68 (0.3) |
| **Brain Cancer** | 118 (0.1) | 25 (0.1) | 25 (0.1) | 30 (0.1) | 38 (0.2) |
| **Leukemia** | 292 (0.3) | 45 (0.2) | 58 (0.3) | 75 (0.3) | 114 (0.5) |

Abbreviations: Q1, first quartile; Q2, second quartile; Q3, third quartile; Q4, fourth quartile.

# Table C. Baseline characteristics of participants by prolonged sedentary behavior quartiles.

| **Characteristic** | **Total** | **Prolonged Sedentary Behavior Quartiles (hours/day)** | | | |
| --- | --- | --- | --- | --- | --- |
|  |  | **Q1 (≤7.0)** | **Q2 (>7.0 – 8.8)** | **Q3 (>8.8 – 10.5)** | **Q4 (>10.5)** |
| *Total N* | 91,292 | 22,823 | 22,824 | 22,822 | 22,823 |
| **Age, years, mean (SD)** | 56.0 (7.8) | 53.8 (7.8) | 55.4 (7.8) | 56.6 (7.7) | 58.4 (7.3) |
| **Sex (%)** |  |  |  |  |  |
| Female | 51,169 (56.0) | 14,716 (64.5) | 13,231 (58.0) | 12,329 (54.0) | 10,893 (47.7) |
| Male | 40,123 (44.0) | 8,107 (35.5) | 9,593 (42.0) | 10,493 (46.0) | 11,930 (52.3) |
| **Deprivation index, mean (SD)** | -1.7 (2.8) | -1.8 (2.8) | -1.8 (2.8) | -1.8 (2.8) | -1.5 (3.0) |
| **Ethnicity (%)** |  |  |  |  |  |
| White | 88,130 (96.9) | 22,009 (96.7) | 22,046 (96.9) | 22,041 (96.9) | 22,034 (97.0) |
| South Asian | 861 (0.9) | 220 (1.0) | 191 (0.8) | 227 (1.0) | 223 (1.0) |
| Black | 774 (0.9) | 208 (0.9) | 210 (0.9) | 166 (0.7) | 190 (0.8) |
| Chinese | 209 (0.2) | 67 (0.3) | 50 (0.2) | 46 (0.2) | 46 (0.2) |
| Mixed | 507 (0.6) | 132 (0.6) | 137 (0.6) | 132 (0.6) | 106 (0.5) |
| Other | 493 (0.5) | 124 (0.5) | 118 (0.5) | 136 (0.6) | 115 (0.5) |
| **Education level (%)** |  |  |  |  |  |
| College or University degree | 39,375 (43.4) | 9,376 (41.3) | 9,863 (43.5) | 10,242 (45.2) | 9,894 (42.5) |
| A levels/AS levels or equivalent | 11,970 (13.2) | 3,129 (13.8) | 3,032 (13.4) | 2,897 (12.8) | 2,912 (12.8) |
| O levels/GCSEs or equivalent | 18,505 (20.4) | 4,930 (21.7) | 4,650 (20.5) | 4,519 (19.9) | 4,406 (19.4) |
| SEs or equivalent | 3,647 (4.0) | 1,260 (5.5) | 971 (4.2) | 768 (3.4) | 644 (2.8) |
| NVQ or HND or HNC or equivalent | 4,873 (5.4) | 1,195 (5.3) | 1,223 (5.4) | 1,145 (5.0) | 1,310 (5.8) |
| Other professional qualifications | 4,538 (5.0) | 1,130 (5.0) | 1,084 (4.8) | 1,169 (5.2) | 1,155 (5.1) |
| None of the above | 7,453 (8.2) | 1,597 (7.0) | 1,774 (7.8) | 1,847 (8.1) | 2,235 (9.9) |
| Prefer not to answer | 382 (0.4) | 92 (0.4) | 83 (0.4) | 92 (0.4) | 115 (0.5) |
| **Never had sugary foods/drinks (%)** | 13,287 (14.6) | 2,993 (13.2) | 3,176 (14.0) | 3,347 (14.8) | 3,771 (16.6) |
| **Dietary intake, mean (SD)** |  |  |  |  |  |
| Fruits and vegetable intake, servings/day | 4.2 (2.3) | 4.4 (2.3) | 4.2 (2.3) | 4.2 (2.2) | 4.1 (2.3) |
| Oil fish intake, times/week | 1.1 (1.0) | 1.1 (1.0) | 1.1 (1.0) | 1.1 (1.0) | 1.1 (1.0) |
| Processed meat intake, times/week | 1.4 (1.4) | 1.3 (1.3) | 1.4 (1.3) | 1.4 (1.4) | 1.5 (1.4) |
| Red meat intake, times/week | 2.1 (1.4) | 2.0 (1.4) | 2.0 (1.3) | 2.1 (1.4) | 2.1 (1.4) |
| **Smoking, mean (SD)** |  |  |  |  |  |
| Never | 52,099 (57.2) | 13,406 (58.9) | 13,168 (57.9) | 13,070 (57.4) | 12,455 (54.7) |
| Previous | 32,593 (35.8) | 7,830 (34.4) | 8,091 (35.6) | 8,188 (36.0) | 8,484 (37.3) |
| Current | 6,354 (7.0) | 1,531 (6.7) | 1,497 (6.6) | 1,507 (6.6) | 1,819 (8.0) |
| **Alcohol consumption, units/week, mean (SD)** | 15.9 (16.7) | 15.6 (16.1) | 16.0 (16.5) | 15.9 (16.5) | 16.0 (17.7) |
| **Multimorbidity count, mean (SD)** | 1.0 (1.1) | 0.8 (1.0) | 0.9 (1.1) | 1.0 (1.1) | 1.2 (1.2) |
| **Time use behaviors, h/day, mean (SD)** |  |  |  |  |  |
| Sleep | 7.2 (1.4) | 7.2 (1.5) | 7.3 (1.4) | 7.3 (1.4) | 6.9 (1.4) |
| LPA | 3.0 (1.0) | 4.0 (1.0) | 3.3 (0.7) | 2.8 (0.6) | 2.1 (0.6) |
| MPA | 0.9 (0.6) | 1.4 (0.8) | 0.9 (0.5) | 0.8 (0.4) | 0.5 (0.3) |
| VPA | 0.1 (0.1) | 0.1 (0.1) | 0.1 (0.1) | 0.1 (0.1) | 0.1 (0.1) |
| **Outcome (%)** |  |  |  |  |  |
| Overall cancer mortality | 1,726 (1.9) | 282 (1.2) | 356 (1.6) | 423 (1.9) | 665 (2.9) |
| Overall cancer incidence | 12,392 (13.6) | 2,449 (10.7) | 2,901 (12.7) | 3,230 (14.2) | 3,812 (16.7) |
| Obesity-related cancer incidence | 4,710 (5.2) | 969 (4.2) | 1,126 (4.9) | 1,199 (5.3) | 1,416 (6.2) |
| T2D-related cancer incidence | 5586 (6.1) | 1,108 (4.9) | 1,279 (5.6) | 1,450 (6.4) | 1,749 (7.7) |

Abbreviations: Q1, first quartile; Q2, second quartile; Q3, third quartile; Q4, fourth quartile; LPA, light physical activity; MPA, moderate physical activity; VPA, vigorous physical activity; BMI, body mass index; WHtR, waist-to-height ratio; WHR, waist-to-hip ratio; WC, waist circumference.

# Table D. Baseline characteristics of participants by interrupted sedentary behavior quartiles.

| **Characteristic** | **Total** | **Interrupted Sedentary Behavior Quartiles (hours/day)** | | | |
| --- | --- | --- | --- | --- | --- |
|  |  | **Q1 (≤2.1)** | **Q2 (>2.1 – 2.9)** | **Q3 (> 2.9 – 3.8)** | **Q4 (> 3.8)** |
| *Total N* | 91,292 | 22,824 | 22,824 | 22,821 | 22,823 |
| **Age, years, mean (SD)** | 56.0 (7.8) | 58.1 (7.5) | 56.4 (7.7) | 55.5 (7.8) | 54.2 (7.9) |
| **Sex (%)** |  |  |  |  |  |
| Female | 51,169 (56.0) | 10,854 (47.6) | 12,249 (53.7) | 13,378 (58.6) | 14,688 (64.4) |
| Male | 40,123 (44.0) | 11,970 (52.4) | 10,575 (46.3) | 9,443 (41.4) | 8,135 (35.6) |
| **Deprivation index, mean (SD)** | -1.7 (2.8) | -1.6 (2.9) | -1.8 (2.8) | -1.8 (2.8) | -1.7 (2.8) |
| **Ethnicity (%)** |  |  |  |  |  |
| White | 88,130 (96.9) | 22,179 (97.6) | 22,062 (97.0) | 22,024 (96.8) | 21,865 (96.1) |
| South Asian | 861 (0.9) | 188 (0.8) | 208 (0.9) | 228 (1.0) | 237 (1.0) |
| Black | 774 (0.9) | 124 (0.5) | 174 (0.8) | 188 (0.8) | 288 (1.3) |
| Chinese | 209 (0.2) | 40 (0.2) | 44 (0.2) | 53 (0.2) | 72 (0.3) |
| Mixed | 507 (0.6) | 99 (0.4) | 118 (0.5) | 130 (0.6) | 160 (0.8) |
| Other | 493 (0.5) | 100 (0.4) | 129 (0.6) | 122 (0.5) | 142 (0.6) |
| **Education level (%)** |  |  |  |  |  |
| College or University degree | 39,375 (43.4) | 9,905 (43.7) | 10,110 (44.6) | 9,671 (42.6) | 9,896 (42.7) |
| A levels/AS levels or equivalent | 11,970 (13.2) | 2,898 (12.8) | 2,821 (12.4) | 3,105 (13.7) | 3,146 (13.9) |
| O levels/GCSEs or equivalent | 18,505 (20.4) | 4,375 (19.3) | 4,625 (20.4) | 4,665 (20.6) | 4,840 (21.3) |
| SEs or equivalent | 3,647 (4.0) | 690 (3.0) | 808 (3.6) | 986 (4.3) | 1,163 (5.1) |
| NVQ or HND or HNC or equivalent | 4,873 (5.4) | 1,310 (5.8) | 1,196 (5.3) | 1,234 (5.4) | 1,133 (5.0) |
| Other professional qualifications | 4,538 (5.0) | 1,161 (5.1) | 1,163 (5.1) | 1,133 (5.0) | 1,081 (4.8) |
| None of the above | 7,453 (8.2) | 2,225 (9.8) | 1,872 (8.3) | 1,797 (7.9) | 1,559 (6.9) |
| Prefer not to answer | 382 (0.4) | 110 (0.5) | 92 (0.4) | 96 (0.4) | 84 (0.4) |
| **Never had sugary foods/drinks (%)** | 13,287 (14.6) | 3,856 (17.0) | 3,327 (14.7) | 3,146 (13.9) | 2,958 (13.0) |
| **Dietary intake, mean (SD)** |  |  |  |  |  |
| Fruits and vegetable intake, servings/day | 4.2 (2.3) | 4.0 (2.3) | 4.2 (2.2) | 4.3 (2.3) | 4.3 (2.3) |
| Oil fish intake, times/week | 1.1 (1.0) | 1.1 (1.0) | 1.1 (1.0) | 1.1 (1.0) | 1.1 (1.0) |
| Processed meat intake, times/week | 1.4 (1.4) | 1.5 (1.4) | 1.4 (1.4) | 1.4 (1.4) | 1.4 (1.3) |
| Red meat intake, times/week | 2.1 (1.4) | 2.1 (1.4) | 2.1 (1.4) | 2.0 (1.4) | 2.0 (1.4) |
| **Smoking, mean (SD)** |  |  |  |  |  |
| Never | 52,099 (57.2) | 12,637 (55.5) | 13,082 (57.4) | 13,056 (57.4) | 13,324 (58.5) |
| Previous | 32,593 (35.8) | 8,359 (36.7) | 8,197 (36.0) | 8,189 (36.0) | 7,848 (34.5) |
| Current | 6,354 (7.0) | 1,755 (7.7) | 1,495 (6.6) | 1,511 (6.6) | 1,593 (7.0) |
| **Alcohol consumption, units/week, mean (SD)** | 15.9 (16.7) | 16.4 (18.0) | 16.0 (16.5) | 15.8 (16.1) | 15.3 (16.2) |
| **Multimorbidity count, mean (SD)** | 1.0 (1.1) | 1.2 (1.2) | 1.0 (1.1) | 1.0 (1.1) | 0.9 (1.0) |
| **Time use behaviors, h/day, mean (SD)** |  |  |  |  |  |
| Sleep | 7.2 (1.4) | 7.1 (1.6) | 7.2 (1.4) | 7.2 (1.4) | 7.2 (1.3) |
| LPA | 3.0 (1.0) | 2.0 (0.5) | 2.8 (0.6) | 3.3 (0.7) | 4.1 (0.9) |
| MPA | 0.9 (0.6) | 0.5 (0.3) | 0.8 (0.4) | 1.0 (0.5) | 1.3 (0.8) |
| VPA | 0.1 (0.1) | 0.1 (0.1) | 0.1 (0.1) | 0.1 (0.1) | 0.1 (0.1) |
| **Outcome (%)** |  |  |  |  |  |
| Overall cancer mortality | 1,726 (1.9) | 660 (2.9) | 422 (1.8) | 355 (1.6) | 289 (1.3) |
| Overall cancer incidence | 12,392 (13.6) | 3,817 (16.7) | 3,206 (14.0) | 2,842 (12.5) | 2,527 (11.0) |
| Obesity-related cancer incidence | 4,710 (5.2) | 1,392 (6.1) | 1,197 (5.2) | 1,086 (4.8) | 1,035 (4.5) |
| T2D-related cancer incidence | 5586 (6.1) | 1,721 (7.5) | 1,430 (6.3) | 1,262 (5.5) | 1173 (5.1) |

Abbreviations: Q1, first quartile; Q2, second quartile; Q3, third quartile; Q4, fourth quartile; LPA, light physical activity; MPA, moderate physical activity; VPA, vigorous physical activity; BMI, body mass index; WHtR, waist-to-height ratio; WHR, waist-to-hip ratio; WC, waist circumference.

# Table E. Association between sedentary behavior and site-specific cancers.

| **Model 1** | | | | | | | |
| --- | --- | --- | --- | --- | --- | --- | --- |
|  | **Overall sedentary behavior**  **(per 1h/day increase)** | | **Prolonged sedentary behavior (per 1h/day increase)** | | | **Interrupted sedentary behavior**  **(per 1h/day increase)** | |
|  | **HR (95% CI)** | **P-value** | **HR (95% CI)** | | **P-value** | **HR (95% CI)** | **P-value** |
| **Esophagus Cancer** | **1.14 (1.04, 1.26)** | **0.007** | **1.08 (1.01, 1.15)** | | **0.026** | 0.94 (0.82, 1.08) | 0.372 |
| **Kidney Cancer** | **1.13 (1.04, 1.23)** | **0.004** | **1.10 (1.04, 1.17)** | | **0.001** | **0.86 (0.80, 0.93)** | **<0.001** |
| **Uterine Cancer** | **1.15 (1.06, 1.24)** | **0.001** | **1.10 (1.05, 1.16)** | | **<0.001** | **0.84 (0.75, 0.94)** | **0.002** |
| **Liver Cancer** | **1.17 (1.04, 1.32)** | **0.011** | **1.09 (1.00, 1.18)** | | **0.046** | 0.95 (0.80, 1.13) | 0.541 |
| **Breast Caner** | **1.06 (1.03, 1.09)** | **<0.001** | **1.05 (1.03, 1.07)** | | **<0.001** | **0.90 (0.87, 0.94)** | **<0.001** |
| **Pancreas Cancer** | **1.14 (1.04, 1.25)** | **0.004** | **1.09 (1.03, 1.16)** | | **0.005** | 0.88 (0.77, 1.00) | 0.056 |
| **Colorectal Cancer** | 1.03 (0.99, 1.08) | 0.105 | **1.03 (1.01, 1.06)** | | **0.013** | **0.92 (0.86, 0.97)** | **0.003** |
| **Bladder Cancer** | 1.04 (0.98, 1.11) | 0.169 | 1.03 (0.99, 1.08) | | 0.097 | 0.93 (0.86, 1.02) | 0.118 |
| **Oral Cancer** | **1.14 (1.04, 1.26)** | **0.006** | **1.11 (1.04, 1.18)** | | **0.001** | **0.81 (0.71, 0.94)** | **0.004** |
| **Lung Cancer** | **1.14 (1.07, 1.21)** | **<0.001** | **1.09 (1.05, 1.14)** | | **<0.001** | **0.86 (0.79, 0.94)** | **0.001** |
| **Melanoma Cancer** | 1.03 (0.97, 1.09) | 0.307 | 1.03 (0.99, 1.07) | | 0.180 | **0.94 (0.90, 1.00)** | **0.035** |
| **Ovary Cancer** | 1.07 (0.97, 1.18) | 0.182 | 1.06 (1.00, 1.13) | | 0.056 | 0.86 (0.75, 0.99) | 0.030 |
| **Prostate Cancer** | 0.99 (0.96, 1.02) | 0.400 | 0.99 (0.97, 1.01) | | 0.228 | 1.03 (0.99, 1.07) | 0.173 |
| **Non-Hodgkin lymphoma** | **1.11 (1.04, 1.18)** | **0.001** | **1.08 (1.03, 1.12)** | | **0.001** | **0.88 (0.81, 0.97)** | **0.006** |
| **Multiple myeloma** | **1.13 (1.02, 1.25)** | **0.022** | **1.17 (1.10, 1.25)** | | **<0.001** | **0.82 (0.71, 0.95)** | **0.009** |
| **Brain Cancer** | 1.03 (0.91, 1.17) | 0.601 | 1.03 (0.95, 1.12) | | 0.455 | 0.93 (0.77, 1.11) | 0.402 |
| **Leukemia** | **1.15 (1.07, 1.25)** | **<0.001** | **1.13 (1.07, 1.19)** | | **<0.001** | **0.76 (0.68, 0.86)** | **<0.001** |
| **Model 2** | | | | | | | |
|  | **Overall sedentary behavior**  **(per 1h/day increase)** | | **Prolonged sedentary behavior**  **(per 1h/day increase)** | | | **Interrupted sedentary behavior**  **(per 1h/day increase)** | |
|  | **HR (95% CI)** | **P-value** | **HR (95% CI)** | **P-value** | | **HR (95% CI)** | **P-value** |
| **Esophagus Cancer** | 1.10 (1.00, 1.22) | 0.060 | 1.05 (0.98, 1.12) | 0.174 | | 0.99 (0.85, 1.14) | 0.848 |
| **Kidney Cancer** | 1.08 (0.99, 1.18) | 0.080 | **1.07 (1.01, 1.14)** | **<0.019** | | **0.85 (0.75, 0.97)** | **0.014** |
| **Uterine Cancer** | **1.12 (1.03, 1.22)** | **0.012** | **1.08 (1.02, 1.14)** | **0.011** | | 0.89 (0.79, 1.01) | 0.064 |
| **Liver Cancer** | 1.12 (0.98, 1.27) | 0.087 | 1.05 (0.97, 1.15) | 0.234 | | 0.99 (0.83, 1.19) | 0.926 |
| **Breast Cancer** | **1.05 (1.02, 1.09)** | **0.002** | **1.04 (1.02, 1.06)** | **<0.001** | | **0.92 (0.88, 0.96)** | **<0.001** |
| **Pancreas Cancer** | **1.11 (1.01, 1.23)** | **0.031** | **1.07 (1.00, 1.14)** | **0.037** | | 0.91 (0.79, 1.04) | 0.172 |
| **Colorectal Cancer** | 1.03 (0.99, 1.07) | 0.186 | **1.03 (1.01, 1.06)** | **0.046** | | **0.93 (0.88, 0.99)** | **0.019** |
| **Bladder Cancer** | 1.03 (0.97, 1.10) | 0.345 | 1.02 (0.98, 1.07) | 0.290 | | 0.96 (0.88, 1.05) | 0.358 |
| **Oral Cancer** | **1.13 (1.02, 1.24)** | **0.018** | **1.10 (1.03, 1.17)** | **0.006** | | **0.84 (0.72, 0.97)** | **0.015** |
| **Lung Cancer** | **1.12 (1.05, 1.19)** | **0.001** | **1.07 (1.03, 1.12)** | **0.001** | | **0.90 (0.82, 0.99)** | **0.030** |
| **Melanoma Cancer** | 1.06 (1.00, 1.12) | 0.056 | **1.04 (1.00, 1.09)** | **0.033** | | 0.93 (0.85, 1.01) | 0.074 |
| **Ovary Cancer** | 1.08 (0.97, 1.19) | 0.162 | 1.07 (1.00, 1.14) | 0.061 | | **0.86 (0.75, 1.00)** | **0.048** |
| **Prostate Cancer** | 0.99 (0.96, 1.02) | 0.459 | 0.99 (0.97, 1.01) | 0.372 | | 1.02 (0.98, 1.06) | 0.404 |
| **Non-Hodgkin lymphoma** | **1.11 (1.04, 1.18)** | **0.002** | **1.08 (1.04, 1.13)** | **<0.001** | | **0.86 (0.78, 0.95)** | **0.002** |
| **Multiple myeloma** | 1.09 (0.98, 1.22) | 0.103 | **1.08 (1.01, 1.16)** | **0.034** | | **0.84 (0.72, 0.98)** | **0.031** |
| **Brain Cancer** | 1.05 (0.92, 1.19) | 0.493 | 1.04 (0.96, 1.13) | 0.375 | | 0.92 (0.76, 1.11) | 0.368 |
| **Leukemia** | **1.15 (1.06, 1.25)** | **0.001** | **1.12 (1.06, 1.19)** | **<0.001** | | **0.77 (0.69, 0.88)** | **<0.001** |

Model 1 adjusted for age, sex, and ethnicity; Model 2 additionally adjusted for deprivation, education, smoking, alcohol intake, intake of sugar, processed meat, red meat, fruit and vegetables, and oil fish.

# Table F. Association between total sedentary behavior and composite cancer outcomes.

| **Model 1** | | | | | | | | |
| --- | --- | --- | --- | --- | --- | --- | --- | --- |
|  | **Q1** | | **Q2** | | **Q3** | | **Q4** | |
|  | **HR (95% CI)** | **P-value** | **HR (95% CI)** | **P-value** | **HR (95% CI)** | **P-value** | **HR (95% CI)** | **P-value** |
| **Overall cancer mortality** | 1 (Reference) | - | 1.06 (0.91, 1.24) | 0.458 | 1.16 (1.00, 1.34) | 0.056 | 1.47 (1.28 , 1.69) | <0.001 |
| **Overall cancer incident** | 1 (Reference) | - | 1.05 (1.00, 1.11) | 0.052 | 1.08 (1.02, 1.14) | 0.005 | 1.14 (1.09, 1.20) | <0.001 |
| **Obesity-related cancer** | 1 (Reference) | - | 1.09 (1.00, 1.19) | 0.052 | 1.15 (1.06, 1.25) | 0.001 | 1.29 (1.19, 1.41) | <0.001 |
| **T2D-related cancers** | 1 (Reference) | - | 1.09 (1.00, 1.18) | 0.043 | 1.20 (1.11, 1.34) | <0.001 | 1.34 (1.24, 1.45) | <0.001 |
| **Model 2** | | | | | | | | |
|  | **Q1** | | **Q2** | | **Q3** | | **Q4** | |
|  | **HR (95% CI)** | **P-value** | **HR (95% CI)** | **P-value** | **HR (95% CI)** | **P-value** | **HR (95% CI)** | **P-value** |
| **Overall cancer mortality** | 1 (Reference) | - | 1.03 (0.88, 1.21) | 0.722 | 1.15 (0.99, 1.34) | 0.069 | 1.38 (1.20, 1.60) | <0.001 |
| **Overall cancer incident** | 1 (Reference) | - | 1.05 (0.99, 1.11) | 0.083 | 1.09 (1.03, 1.15) | 0.003 | 1.13 (1.07, 1.20) | <0.001 |
| **Obesity-related cancer** | 1 (Reference) | - | 1.08 (0.98, 1.18) | 0.104 | 1.17 (1.07, 1.28) | 0.001 | 1.26 (1.15, 1.38) | <0.001 |
| **T2D-related cancers** | 1 (Reference) | - | 1.06 (0.98, 1.16) | 0.141 | 1.20 (1.11, 1.30) | <0.001 | 1.28 (1.18, 1.39) | <0.001 |

Abbreviations: Q1, first quartile; Q2, second quartile; Q3, third quartile; Q4, fourth quartile.

Model 1 adjusted for age, sex, and ethnicity; Model 2 additionally adjusted for deprivation, education, smoking, alcohol intake, intake of sugar, processed meat, red meat, fruit and vegetables, and oil fish.

Obesity-related cancer including esophagus cancer, liver cancer, kidney cancer, myeloma, pancreatic cancer, colorectal cancer, gallbladder cancer, breast cancer, ovarian cancer, and thyroid cancer.

# Table G. Sensitivity analysis of the association between sedentary behavior and composite cancer risk adjusted for BMI and morbidity count.

|  | **Overall sedentary behavior**  **(per 1h/day increase)** | | **Prolonged sedentary behavior**  **(per 1h/day increase)** | | **Interrupted sedentary behavior**  **(per 1h/day increase)** | |
| --- | --- | --- | --- | --- | --- | --- |
|  | **HR (95% CI)** | **P-value** | **HR (95% CI)** | **P-value** | **HR (95% CI)** | **P-value** |
| **Overall cancer mortality** | 1.07 (1.04, 1.11) | <0.001 | 1.05 (1.04, 1.07) | <0.001 | 0.85 (0.80, 0.86) | <0.001 |
| **Overall cancer incidence** | 1.02 (1.01, 1.04) | 0.001 | 1.02 (1.01, 1.03) | <0.001 | 0.95 (0.93, 0.96) | <0.001 |
| **Obesity-related cancer** | 1.05 (1.03, 1.07) | <0.001 | 1.07 (1.05, 1.10) | <0.001 | 0.92 (0.90, 0.95) | <0.001 |
| **T2D-related cancer** | 1.05 (1.03, 1.07) | <0.001 | 1.04 (1.03, 1.06) | <0.001 | 0.92 (0.89, 0.95) | <0.001 |

Model adjusted for age, sex, and ethnicity, deprivation, education, smoking, alcohol intake, sugar intake, processed meat, red meat, fruit and vegetables, oil fish, BMI, morbidity count.

Obesity-related cancer including esophagus cancer, liver cancer, kidney cancer, myeloma, pancreatic cancer, colorectal cancer, gallbladder cancer, breast cancer, ovarian cancer, and thyroid cancer.

T2D-related cancers including thyroid cancer, breast cancer, liver cancer, pancreatic cancer, endometrial cancer, esophagus cancer, colorectal cancer, kidney cancer, gallbladder cancer, ovarian cancer, non-Hodgkin lymphoma, leukemia, and bladder cancer.

**Table H. Association between intensity of physical activity and composite cancer outcomes.**

|  | **LPA (per 1 h/d increase)** | | **MPA (per 30 min/d increase)** | | **VPA (per 5 min/d increase)** | |
| --- | --- | --- | --- | --- | --- | --- |
|  | **HR (95% CI)** | **P-value** | **HR (95% CI)** | **P-value** | **HR (95% CI)** | **P-value** |
| **Overall cancer mortality** | 0.81 (0.76 , 0.86) | <0.001 | 0.83 (0.78 , 0.87) | <0.001 | 0.77 (0.72, 0.83) | <0.001 |
| **Overall cancer incidence** | 0.92 (0.90, 0.94) | <0.001 | 0.96 (0.94, 0.98) | <0.001 | 0.95 (0.93, 0.97) | <0.001 |
| **Obesity-related cancer** | 0.88 (0.85, 0.92) | <0.001 | 0.95 (0.92, 0.98) | <0.001 | 0.89 (0.86, 0.93) | <0.001 |
| **T2D-related cancer** | 0.87 (0.85, 0.90) | <0.001 | 0.93 (0.90, 0.95) | <0.001 | 0.87 (0.84, 0.90) | <0.001 |

Model adjusted for age, sex, ethnicity, deprivation, education, smoking, alcohol intake, intake of sugar, processed meat, red meat, fruit and vegetables, and oil fish.

Abbreviations: LPA, light physical activity; MPA, moderate physical activity; VPA, vigorous physical activity.

# Table I. Association between intensity of physical activity and site-specific cancers.

|  | **LPA (1 hour/day)** | | **MPA (30 mins/day)** | | **VPA (5 mins/day)** | |
| --- | --- | --- | --- | --- | --- | --- |
|  | **HR (95% CI)** | **P-value** | **HR (95% CI)** | **P-value** | **HR (95% CI)** | **P-value** |
| **Esophagus Cancer** | 0.94 (0.80, 1.12) | 0.509 | 0.98 (0.84, 1.14) | 0.774 | **0.76 (0.62, 0.94)** | **0.011** |
| **Kidney Cancer** | **0.77 (0.66 , 0.90)** | **0.001** | 0.92 (0.80, 1.05) | 0.204 | **0.80 (0.67, 0.95)** | **0.012** |
| **Uterine Cancer** | 0.92 (0.80, 1.06) | 0.263 | **0.85 (0.75, 0.97)** | **0.019** | **0.79 (0.66, 0.95)** | **0.013** |
| **Liver Cancer** | 0.86 (0.69, 1.07) | 0.178 | 0.97 (0.80, 1.17) | 0.718 | **0.67 (0.49, 0.92)** | **0.012** |
| **Breast Caner** | **0.87 (0.82, 0.92)** | **<0.001** | 0.98 (0.94, 1.02) | 0.372 | **0.92 (0.87, 0.97)** | **0.003** |
| **Pancreas Cancer** | 0.96 (0.82, 1.13) | 0.630 | **0.85 (0.72, 0.99)** | **0.043** | 0.85 (0.71, 1.02) | 0.085 |
| **Colorectal Cancer** | 0.93 (0.86, 1.00) | 0.041 | 0.95 (0.89, 1.01) | 0.108 | **0.85 (0.79, 0.92)** | **<0.001** |
| **Bladder Cancer** | 0.94 (0.85, 1.05) | 0.264 | **0.84 (0.76, 0.93)** | **0.001** | **0.86 (0.77, 0.96)** | **0.008** |
| **Oral Cancer** | 0.86 (0.73, 1.02) | 0.090 | 0.91 (0.78, 1.06) | 0.220 | 0.88 (0.75, 1.03) | 0.102 |
| **Lung Cancer** | **0.88 (0.78, 0.98)** | **0.024** | 0.84 (0.75, 0.94) | 0.002 | **0.76 (0.65, 0.88)** | **<0.001** |
| **Melanoma Cancer** | 0.92 (0.83, 1.02) | 0.101 | 0.93 (0.85, 1.01) | 0.098 | 1.00 (0.92, 1.08) | 0.937 |
| **Ovary Cancer** | 0.85 (0.72, 1.01) | 0.065 | 0.89 (0.77, 1.03) | 0.131 | 0.80 (0.65, 0.99) | 0.039 |
| **Prostate Cancer** | 0.99 (0.94, 1.05) | 0.798 | **1.05 (1.01, 1.10)** | **0.026** | 1.03 (0.99, 1.07) | 0.110 |
| **Non-Hodgkin lymphoma** | **0.84 (0.75 , 0.94)** | **0.002** | **0.90 (0.82, 1.00)** | **0.047** | 0.91 (0.82, 1.01) | 0.067 |
| **Multiple myeloma** | 0.89 (0.74, 1.06) | 0.191 | **0.76 (0.63, 0.92)** | **0.005** | 0.89 (0.74, 1.06) | 0.181 |
| **Brain Cancer** | **0.76 (0.61, 0.96)** | **0.019** | 1.06 (0.90, 1.26) | 0.459 | 0.87 (0.70, 1.08) | 0.209 |
| **Leukemia** | **0.78 (0.67, 0.90)** | **0.001** | **0.77 (0.66, 0.89)** | **<0.001** | **0.78 (0.66, 0.92)** | **0.004** |

Model adjusted for age, sex, ethnicity, deprivation, education, smoking, alcohol intake, intake of sugar, processed meat, red meat, fruit and vegetables, and oil fish.

Abbreviations: LPA, light physical activity; MPA, moderate physical activity; VPA, vigorous physical activity.

# Table J. Hazard ratios for site specific cancer outcomes associated with replacing type-specific SB with other SB and physical activity in isotemporal substitution models.

|  | **Esophagus Cancer** | | **Kidney Cancer** | | **Uterine Cancer** | | **Liver Cancer** | |
| --- | --- | --- | --- | --- | --- | --- | --- | --- |
|  | **HR (95% CI)** | **P-value** | **HR (95% CI)** | **P-value** | **HR (95% CI)** | **P-value** | **HR (95% CI)** | **P-value** |
| **Replacing prolonged SB with:** |  |  |  |  |  |  |  |  |
| Interrupted SB (1h/d) | 1.10 (0.81, 1.49) | 0.500 | 1.11 (0.84, 1.47) | 0.500 | 0.95 (0.72, 1.25) | 0.700 | **1.54 (1.03, 2.30)** | **0.037** |
| LPA (1h/d) | 0.90 (0.65, 1.23) | 0.500 | **0.73 (0.54, 0.99)** | **0.040** | 1.02 (0.77, 1.35) | >0.900 | **0.61 (0.39, 0.94)** | **0.027** |
| MPA (30min/d) | 1.03 (0.85, 1.25) | 0.800 | 0.97 (0.82, 1.16) | 0.800 | 0.92 (0.77, 1.09) | 0.300 | 0.93 (0.72, 1.19) | 0.600 |
| VPA (5min/d) | **0.75 (0.60, 0.94)** | **0.013** | 0.83 (0.70, 1.00) | 0.051 | 0.84 (0.69, 1.02) | 0.072 | **0.68 (0.49, 0.95)** | **0.023** |
| **Replacing interrupted SB with:** |  |  |  |  |  |  |  |  |
| Prolonged SB (1h/d) | 1.06 (0.95, 1.19) | 0.300 | 1.00 (0.91, 1.10) | >0.900 | 1.08 (0.97, 1.20) | 0.140 | 1.00 (0.87, 1.14) | >0.900 |
| LPA (1h/d) | 1.07 (0.83, 1.38) | 0.600 | **0.80 (0.64, 1.00)** | **0.045** | 1.09 (0.88, 1.36) | 0.400 | 0.89 (0.66, 1.21) | 0.500 |
| MPA (30min/d) | 1.12 (0.94, 1.34) | 0.200 | 1.01 (0.87, 1.18) | 0.900 | 0.96 (0.82, 1.13) | 0.600 | 1.10 (0.89, 1.36) | 0.400 |
| VPA (5min/d) | **0.75 (0.60, 0.94)** | **0.013** | **0.83 (0.69, 1.00)** | **0.046** | 0.84 (0.69, 1.02) | 0.079 | **0.66 (0.48, 0.93)** | **0.017** |
|  | **Breast Cancer** | | **Pancreas Cancer** | | **Colorectal Cancer** | | **Bladder Cancer** | |
|  | **HR (95% CI)** | **P-value** | **HR (95% CI)** | **P-value** | **HR (95% CI)** | **P-value** | **HR (95% CI)** | **P-value** |
| **Replacing prolonged SB with:** |  |  |  |  |  |  |  |  |
| Interrupted SB (1h/d) | 1.04 (0.94, 1.15) | 0.400 | 0.90 (0.67, 1.21) | 0.500 | 0.94 (0.83, 1.07) | 0.400 | **1.23 (1.01, 1.50)** | **0.036** |
| LPA (1h/d) | **0.84 (0.76, 0.94)** | **0.001** | 1.12 (0.82, 1.52) | 0.500 | 1.01 (0.88, 1.15) | >0.900 | 0.83 (0.68, 1.03) | 0.4089 |
| MPA (30min/d) | 1.00 (0.95, 1.06) | >0.900 | 0.91 (0.74, 1.08) | 0.200 | 1.02 (0.94, 1.11) | 0.600 | **0.80 (0.70, 0.92)** | **0.002** |
| VPA (5min/d) | **0.94 (0.89 , 0.99)** | **0.023** | 0.93 (0.81, 1.08) | 0.300 | **0.85 (0.78 , 0.93)** | **<0.001** | 0.91 (0.81, 1.01) | 0.110 |
| **Replacing interrupted SB with:** |  |  |  |  |  |  |  |  |
| Prolonged SB (1h/d) | 1.01 (0.97, 1.04) | 0.700 | 1.10 (0.98, 1.23) | 0.110 | 1.01 (0.96, 1.06) | 0.600 | 0.97 (0.90, 1.04) | 0.400 |
| LPA (1h/d) | **0.89 (0.82, 0.96)** | **0.002** | 1.18 (0.92, 1.52) | 0.200 | 0.98 (0.88, 1.08) | 0.600 | 0.95 (0.82, 1.11) | 0.600 |
| MPA (30min/d) | 1.02 (0.97, 1.08) | 0.400 | 0.95 (0.78, 1.15) | 0.600 | 1.01 (0.94, 1.09) | 0.800 | **0.84 (0.74, 0.96)** | **0.010** |
| VPA (5min/d) | **0.94 (0.88 , 0.99)** | **0.021** | 0.90 (0.75, 1.09) | 0.3 00 | **0.85 (0.79, 0.93)** | **<0.001** | 0.90 (0.80, 1.01) | 0.079 |
|  | **Oral Cancer** | | **Lung Cancer** | | **Melanoma Cancer** | | **Ovary Cancer** | |
|  | **HR (95% CI)** | **P-value** | **HR (95% CI)** | **P-value** | **HR (95% CI)** | **P-value** | **HR (95% CI)** | **P-value** |
| **Replacing prolonged SB with:** |  |  |  |  |  |  |  |  |
| Interrupted SB (1h/d) | 0.78 (0.58, 1.04) | 0.093 | 1.15 (0.93, 1.42) | 0.200 | 1.00 (0.84, 1.20) | >0.900 | 0.98 (0.71, 1.35) | 0.900 |
| LPA (1h/d) | 1.10 (0.82, 1.48) | 0.500 | 0.83 (0.66, 1.04) | 0.100 | 0.93 (0.77, 1.12) | 0.500 | 0.90 (0.65, 1.26) | 0.600 |
| MPA (30min/d) | 1.06 (0.88, 1.27) | 0.600 | **0.86 (0.75, 0.99)** | **0.041** | 0.93 (0.83, 1.04) | 0.200 | 0.97 (0.80, 1.17) | 0.700 |
| VPA (5min/d) | 0.90 (0.76, 1.06) | 0.200 | **0.81 (0.69, 0.95)** | **0.011** | 1.02 (0.94, 1.11) | 0.600 | 0.84 (0.68, 1.05) | 0.130 |
| **Replacing interrupted SB with:** |  |  |  |  |  |  |  |  |
| Prolonged SB (1h/d) | 1.11 (0.99, 1.24) | 0.077 | 1.05 (0.97, 1.14) | 0.200 | 1.04 (0.97, 1.12) | 0.200 | 1.00 (0.09, 1.12) | >0.900 |
| LPA (1h/d) | 1.04 (0.81, 1.33) | 0.700 | 1.01 (0.85, 1.20) | 0.900 | 1.00 (0.86, 1.16) | >0.900 | 0.89 (0.70, 1.13) | 0.300 |
| MPA (30min/d) | 1.05 (0.88, 1.25) | 0.600 | 0.95 (0.83, 1.08) | 0.400 | 0.96 (0.87, 1.07) | 0.500 | 0.96 (0.81, 1.14) | 0.600 |
| VPA (5min/d) | 0.91 (0.77, 1.07) | 0.300 | **0.81 (0.69, 0.95)** | **0.010** | 1.02 (0.95, 1.11) | 0.600 | 0.84 (0.68, 1.05) | 0.130 |
|  | **Prostate Cancer** | | **Non-Hodgkin lymphoma** | | **Multiple myeloma** | | **Brain Cancer** | |
|  | **HR (95% CI)** | **P-value** | **HR (95% CI)** | **P-value** | **HR (95% CI)** | **P-value** | **HR (95% CI)** | **P-value** |
| **Replacing prolonged SB with:** |  |  |  |  |  |  |  |  |
| Interrupted SB (1h/d) | 1.03 (0.94, 1.12) | 0.500 | 0.97 (0.79, 1.19) | 0.800 | 0.93 (0.66 , 1.29) | 0.6 00 | 1.24 (0.83, 1.84) | 0.300 |
| LPA (1h/d) | 0.95 (0.87, 1.04) | 0.300 | 0.89 (0.72, 1.09) | 0.300 | 1.02 (0.72, 1.44) | >0.900 | **0.63 (0.41, 0.96)** | **0.031** |
| MPA (30min/d) | 1.04 (0.98, 1.10) | 0.200 | 0.96 (0.84, 1.09) | 0.500 | 0.81 (0.64, 1.03) | 0.081 | 1.06 (0.85, 1.32) | 0.600 |
| VPA (5min/d) | 1.02 (0.98, 1.06) | 0.300 | 0.94 (0.85, 1.05) | 0.300 | 0.97 (0.81, 1.14) | 0.700 | 0.87 (0.69, 1.09) | 0.200 |
| **Replacing interrupted SB with:** |  |  |  |  |  |  |  |  |
| Prolonged SB (1h/d) | 1.00 (0.96, 1.03) | 0.800 | 1.08 (1.00, 1.17) | 0.058 | 1.01 (0.90, 1.14) | 0.800 | 0.98 (0.85, 1.13) | 0.800 |
| LPA (1h/d) | 0.97 (0.90, 1.04) | 0.400 | 0.97 (0.82, 1.15) | 0.700 | 0.97 (0.75, 1.26) | 0.800 | 0.73 (0.54, 1.00) | 0.052 |
| MPA (30min/d) | 1.05 (0.99, 1.10) | 0.100 | 1.01 (0.89, 1.14) | >0.900 | **0.80 (0.64, 0.99)** | **0.043** | 1.13 (0.93, 1.37) | 0.200 |
| VPA (5min/d) | 1.02 (0.98, 1.06) | 0.300 | 0.95 (0.85, 1.05) | 0.300 | 0.97 (0.82, 1.15) | 0.700 | 0.86 (0.68, 1.09.) | 0.200 |
|  | **Leukemia** | |  |  |  |  |  |  |
|  | **HR (95% CI)** | **P-value** |  |  |  |  |  |  |
| **Replacing prolonged SB with:** |  |  |  |  |  |  |  |  |
| Interrupted SB (1h/d) | 0.89 (0.68, 1.17) | 0.400 |  |  |  |  |  |  |
| LPA (1h/d) | 0.93 (0.70, 1.23) | 0.600 |  |  |  |  |  |  |
| MPA (30min/d) | 0.89 (0.74, 1.07) | 0.200 |  |  |  |  |  |  |
| VPA (5min/d) | 0.87 (0.74, 1.03) | 0.100 |  |  |  |  |  |  |
| **Replacing interrupted SB with:** |  |  |  |  |  |  |  |  |
| Prolonged SB (1h/d) | 1.04 (0.95, 1.14) | 0.400 |  |  |  |  |  |  |
| LPA (1h/d) | 0.90 (0.73, 1.10) | 0.300 |  |  |  |  |  |  |
| MPA (30min/d) | 0.88 (0.74, 1.04) | 0.130 |  |  |  |  |  |  |
| VPA (5min/d) | 0.87 (0.74, 1.03) | 0.120 |  |  |  |  |  |  |

Model adjusted for age, sex, ethnicity, deprivation, education, smoking, alcohol intake, intake of sugar, processed meat, red meat, fruit and vegetables, and oil fish.

Abbreviations: LPA, light physical activity; MPA, moderate physical activity; VPA, vigorous physical activity; ISB, Interrupted sedentary behavior.

# Table K. Association between sedentary behavior and incident of composite cancer, excluding first two years of follow-up.

|  | **Total Sedentary behavior**  **(per 1h/day increase)** | | **Prolonged sedentary behavior**  **(per 1h/day increase)** | | **Interrupted sedentary behavior**  **(per 1h/day increase)** | |
| --- | --- | --- | --- | --- | --- | --- |
|  | **HR (95% CI)** | **P-value** | **HR (95% CI)** | **P-value** | **HR (95% CI)** | **P-value** |
| **Overall cancer mortality** | 1.10 (1.06, 1.14) | <0.001 | 1.08 (1.06, 1.11) | <0.001 | 0.84 (0.80, 0.88) | <0.001 |
| **Overall cancer incidence** | 1.03 (1.01, 1.04) | <0.001 | 1.03 (1.02, 1.04) | <0.001 | 0.94 (0.92, 0.96) | <0.001 |
| **Obesity-related cancer** | 1.05 (1.03, 1.08) | <0.001 | 1.04 (1.03, 1.06) | <0.001 | 0.91 (0.89, 0.94) | <0.001 |
| **T2D-related cancer** | 1.07 (1.04, 1.09) | <0.001 | 1.05 (1.04, 1.07) | <0.001 | 0.91 (0.88, 0.93) | <0.001 |

Model adjusted for age, sex, ethnicity, deprivation, education, smoking, alcohol intake, intake of sugar, processed meat, red meat, fruit and vegetables, and oil fish.

Obesity-related cancer including esophagus cancer, liver cancer, kidney cancer, myeloma, pancreatic cancer, colorectal cancer, gallbladder cancer, breast cancer, ovarian cancer, and thyroid cancer.

T2D-related cancers including thyroid cancer, breast cancer, liver cancer, pancreatic cancer, endometrial cancer, esophagus cancer, colorectal cancer, kidney cancer, gallbladder cancer, ovarian cancer, non-Hodgkin lymphoma, leukemia, and bladder cancer.

# Fig A. Non-linear association between intensity of physical activity and composite cancer outcomes.


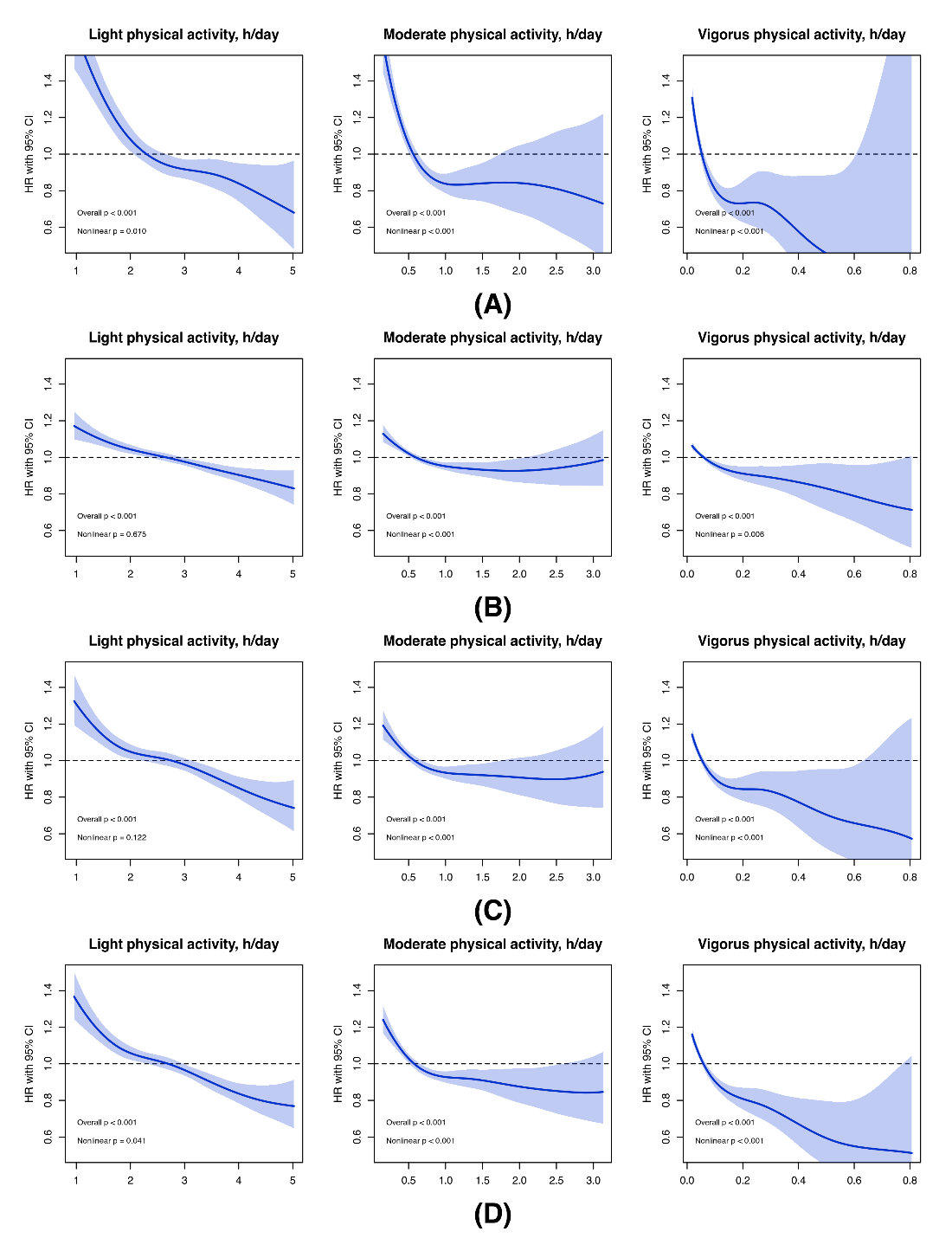


All models were adjusted for age, sex, and ethnicity, deprivation, education, smoking, intake of alcohol, sugar, processed meat, red meat, fruit and vegetables, oily fish. Obesity-related cancer including esophagus cancer, liver cancer, kidney cancer, myeloma, pancreatic cancer, colorectal cancer, gallbladder cancer, breast cancer, ovarian cancer, and thyroid cancer. T2D-related cancers including thyroid cancer, breast cancer, liver cancer, pancreatic cancer, endometrial cancer, esophagus cancer, colorectal cancer, kidney cancer, gallbladder cancer, ovarian cancer, non-Hodgkin lymphoma, leukemia, and bladder cancer.

Panels (A)(B)(C)(D) represent the association between overall cancer mortality, the incidence of overall, obesity-related, and T2D-related cancers, respectively.
