# Supplementary material for: Accelerometry-measured prolonged and interrupted sedentary behavior and cancer incidence and mortality: A cohort study of 91,292 UK Biobank participants
Source: PLoS Med. 2026 Jul 2;23(7):e1004767. doi: 10.1371/journal.pmed.1004767 (PMC13327276; doi:10.1371/journal.pmed.1004767)
Supplement: Attachment — Submitted filename: Response letter_plos med.docx [file pmed.1004767.s003.docx]

Comments from the academic editor:

Accelerometry-measured prolonged and interrupted sedentary behavior and cancer incidence and mortality: A prospective cohort study of 92,354 UK Biobank participants

This manuscript examines the association of interrupted for prolonged sedentary behaviors (SB) on risk of incident cancer and cancer mortality. Through isotemporal models, the authors show that prolonged and interrupted SB have diverging associations with risk, with prolonged SB associated with increased risk and interrupted SB associated with decreased risk, all while holding levels of other activity constant. This provides an important proof of concept for the SB construct—it’s not just your total physical activity level, but whether your activity is used to break up bouts of long sitting. That’s a distinct scientific contribution that sets the paper apart from others in the literature.

In general, the analysis is well conducted and thoughtful, getting at key concepts more directly than prior papers. The presentation of the methods and results for the isotemporal models still need work, though, as the paper is sometimes hard to follow. I have several suggestions that may help improve readability and strengthen the science.

Major comments
1. “Replacing with” and “substituting with”: The manuscript uses these two phrases interchangeably. While this is grammatically acceptable, “substituting X with Y” is inherently ambiguous: most readers will interpret it correctly as “replacing X with Y,” but a nontrivial minority may read it as “adding X in place of Y.” Using two different phrases to express the same idea compounds this ambiguity. In contrast, “replacing with” is unambiguous. I recommend eliminating “substituting with” and using “replacing with” consistently throughout for greater clarity.

RESPONSE: As suggested “substituting with” now has been changed to “replacing with” in the manuscript.

2. Methods and isotemporal substitution model (ISMs): The analysis examined two distinct ISMs. The authors should present each individually in the methods and define the meaning of terms specific to each model separately. This will substantially clarify the text on B1 to B5, which is presently incomplete (no description of B5) and logically incorrect (the effects describe replacing a specific kind of SB, not just any SB).

RESPONSE: Thank you for the suggestion. The two ISM models have been re-written separately in the Methods section p15-16.

3. Editing: The quality of editing declines toward the end of the manuscript, with several copy errors, some awkward phrasings, a few incomplete thoughts, and occasional non sequiturs. Given the complexity of the models, the added uncertainty about the text’s accuracy substantially increases the cognitive burden on readers. The authors should check the document carefully.

RESPONSE: Thank you for careful reading of our paper. We apologize for the typos and language errors. We have revised as suggested below and carefully proofread the paper.

Some phrases that need fixing:

“Table 2 shows the associations between associations between..” (p.13)

RESPONSE: The duplicate “associations between” have been removed.

“both total sedentary time and sedentary bout duration…it had” (p.16—what is “it”?)

RESPONSE: The sentence now has been clarified.

“which consist of the results of overall cancer incident with our study” (p.17)

RESPONSE: The sentence now has been edited.

“which still partly had the similar individual results as our study” (p.17)

RESPONSE: The sentence now has been edited.

“The ISM results from this study consistent with” (p.17)

RESPONSE: The sentence now has been edited.

“Additionally, the dose-response association for risk of cancer mortality when total sedentary time and mean sedentary bout length were expressed prolongedly” (p.17)

RESPONSE: The sentence now has been edited.

“This sheds light on the mechanism how SB could influence health outcomes – both by itself accumulated in a prolonged manner, as well as to replace PA.” (p.17-18—I’m not sure what the point is here. Authors should make the sentence direct and punchy—how exactly does it shed light?)

RESPONSE: The sentence now has been expanded.

“In particular, it has been shown that device measured total SB was associated with endothelial dysfunction and lowgrade inflammation, adjusted for PA (35). (p.18—is this relevant given this paper’s focus on “total SB”? Especially considering the next sentence..)

RESPONSE: The sentence now has been edited.

“But no significant association with sedentary breaks or average length of sedentary bouts, potentially due to the different operationalization of interrupting SB, as well as their smaller sample size (n=2363).” (p.18—the sentence lacks a verb/predicate)

RESPONSE: The sentence now has been edited; a verb has been added.

“even when body fat is not a factor” (p.18—this is immediately followed by a sentence about why fat is important. Perhaps this meant to say body fat percent? Regardless, that’s not the entry point I would use for this topic)

RESPONSE: Thank you for pointing out the inconsistencies in the description. We have now rewritten that paragraph for clarity.

“A particular concern is the accumulation of ectopic fat…” (p.18—how specifically is this a concern with respect to SB-cancer associations? Are SBs associated with ectopic fat? The passively constructed argument here makes it hard to tell whether there is a logically complete argument).

RESPONSE: The sentence has been edited and clarified.

“Insulin and insulin-like growth factor I (IGF-I) are anabolic hormones that play critical roles in regulating glucose metabolism, cell growth, apoptosis, and angiogenesis (32). Excessive activation of these hormones, along with their associated binding proteins (IGFBP-1 through -6) and signaling pathways, has been linked to an increased risk of several cancers, including breast, prostate, and colorectal cancer (32).” (p.18—Are these factors linked with SB? To what extent? Specifically with prolonged sitting?)

RESPONSE: The sentence has been edited and clarified.

“Second, it used validated, machine learning-informed, approach to derive the SB which is more accurate than the questionnaire-based study” (p.19)

RESPONSE: The sentence has been edited

“which has been shown using a different analysis” (p.20—as phrased, this will make the reader think the point is the use of a different model, not the use of the same study population. Authors should amend).

RESPONSE: The sentence has been clarified.

4. Comorbidities: The lack of adjustment for comorbidities, e.g. diabetes, will be a major concern for many readers. I appreciate the sensitivity analysis (and the point about “confounders or mediators”) but the methods and results should include more details about this analysis, since it is rather important. Authors should describe the construction of the comorbidity count in the main text (including the list of comorbidities considered and any weighting) and provide more details on the results.

RESPONSE: The long term conditions included were based on the landmark 2012 Lancet paper on multimorbidity by Barnet et al. The list of the conditions and relevant references are added.

Minor comments

5. Abstract: In the methods, covariate adjustments should be noted. In the results, authors should aim for parallel construction of the last two sentences, and use the correct subscripts after HR.

RESPONSE: The Abstract now has been added ‘after adjusted covariate’ and edited for parallel construction and subscripts after HR.

6. Introduction: Authors should briefly describe the biological rationale. I recall interventional studies of volunteers with prolonged sitting that examined levels of several biomarkers (e.g. Dunstan et al., 2012; Peddie et al., 2013). Perhaps these are relevant?

RESPONSE: Biological rationale has been added.

7. Cancer endpoints: Esophageal—please clarify the percent that are esophageal adenocarcinoma. Multiple myeloma—this is a general code for plasma cell neoplasms (most of which are multiple myeloma). I’m fine with using this code, but a minor clarification is in order. Leukemia—I’m fine with this coding, but studies increasingly split this by cell lineage, i.e. myeloid or lymphoid. Multiple melanoma—delete the word multiple here.

RESPONSE: Thank you for spotting the nuances. We have added the %s for multiple myeloma, and clarified the C90 code included other plasma cell neoplasms. Unfortunately we do not have power to separate the leukemia by cell lineage, or the information of esophageal adenocarcinoma, which are acknowledged in the limitations. We have deleted ‘multiple’ for melanoma.

8. Discussion: Consistent with the concerns outlined in the “Editing” section above, the Mechanisms section should be revised extensively to improve clarity.

RESPONSE: We have now edited the Mechanisms for clarity.

9. Tables and figures: Table 1: Values flipped for sugar intake in the total column. Smoking should be added, also alcohol. All items should be listed in the same order as Footnote to Table 2 (with variables for sensitivity analysis last, as currently done). Table 4. The “ratio of HR” is space-efficient, but most readers will misunderstand it. I would either present the full set of results or just use the p-values for interaction. The title needs to indicate whether this is the ISM or non-ISM model. If the latter, please include before any ISM models are presented. The word “obesity” should be replaced by “adiposity” (obesity is specific to BMI).

RESPONSE: Thank you for the suggestions. We have now corrected sugar intake, and added smoking and alcohol, reworded ‘obesity’ to be ‘adiposity’, clarified in the table title the models being used. However, we prefer not to present the p-value for interactions only as we think the magnitude of interaction is also important. But we understand that readers could misunderstand it and added an explanatory footnote.

Reviewer #1: This manuscript describes results from an analysis of the UK biobank on the association between interrupted and prolonged SB on cancer. My comments are:
- A Lot of the hazard ratios are rather small and are significant mostly as a result of a large sample size and therefore the concept of clinically significant becomes more important. Are these hazard ratios clinically significant?

RESPONSE: We appreciate the thoughtful comment. But some key findings, e.g. replacing prolonged sedentary time by light PA, showed sizable risk reduction: 12% for cancer mortality and 10-11% for obesity/diabetes related cancer incidence. We also want to highlight that even with smaller effect sizes, the potential impact could be vast at the population level. Nonetheless, we have included a small discussion to note that some effect sizes to be rather small and careful cost-effectiveness analysis would be required if the interventions to change these behaviors are costly.

- "The optimal strategy for replacing prolonged sedentary behavior will vary based on individual ability and preferences." Is that something that your analysis shows?

RESPONSE: Indeed, our analysis does not directly evaluate individual preferences or determine an “optimal” behavioral strategy. We have therefore removed the text to avoid overstating this interpretation.

- "Participants with insufficient wear time (<72 hours), missing data, or poor device calibration were excluded." How many were these, especially the missing data part? Complete case analysis is not recommended unless the amount of missing data is very low and methods such as imputation should be used.

RESPONSE: Apologies for the confusion. The ‘missing’ data in that sentence refers to people not participating in the accelerometer sub-study in the UK Biobank, rather than missing data after they participated. We have rewritten that part to avoid confusion.

- Is the variable Prolonged PA measured once or is it time-dependent?

RESPONSE: Prolonged PA was ascertained from the 7-day accelerometer measurements and therefore defined using all the available data from those 7 days. There are no repeated measures on accelerometer and the prolonged PA was treated as a constant in analysis.

- "Coefficients β₁ to β₅ represented the effects of substituting SB with sleep, LPA, MPA, and VPA  respectively, while holding total time constant." I have a hard time understanding how the "intervention" variable was defined. Is it a categorical variable with the levels in the equation on page 11. I assume people can satisfy more than one of these levels. How were they classified? More generally, I think a better description of the intervention would be helpful.

RESPONSE: We appreciate the reviewer’s comment and agree that our previous wording made the isotemporal models sound like they involved a categorical intervention variable. This was not the case. We have revised the Methods to clarify that participants were not classified into mutually exclusive replacement groups. Instead, all movement behaviors were modeled as continuous daily time variables, and the “replacement” interpretation arises from the isotemporal framework by holding total time constant and omitting one behavior from the model. We now describe each isotemporal substitution model separately and state explicitly what each coefficient represents.

- "Table 3 shows the risks of composite cancer outcomes when one type of SB was replaced by the other type of SB, LPA, MPA, or VPA." I don't think this is the correct interpretation of hazard ratios and risk and hazard ratios seem to be used to mean the same thing throughout, which is not exactly correct either.

RESPONSE: We have used ‘risk’ because for rarer events like cancer, hazard and risk are similar, and that even though the quantity could be different, the direction between risk and hazard (an instantaneous rate) should be the same. We have made this assumption for interpretation explicit in the Methods. We would also like to emphasize that the hazard ratio in this isotemporal substitution model is representing the relative change in hazard associated with replacing one activity with another. A reference for the method is added in Methods.

Mekary R.A., Willett W.C., Hu F.B., Ding E.L. Isotemporal substitution paradigm for physical activity epidemiology and weight change. Am. J. Epidemiol. 2009;170:519–527. doi: 10.1093/aje/kwp163

Reviewer #2: Dear authors,

Thank you for giving me the opportunity to review this interesting manuscript.

In their study, the authors investigated the cancer risk associated with prolonged and interrupted sedentary behaviour. They used prospective data from the UK Biobank and a random forest classifier to quantify sedentary behaviour from wrist-worn accelerometer data. The manuscript provides a thorough analysis, including a detailed sensitivity analysis. The findings of this study advance our understanding of the detrimental effects of uninterrupted sedentary behaviour compared to breaking up sitting. The manuscript is well written and follows a clear structure.

I have a few comments which I hope will help the authors further improve it. I must acknowledge, however, that I have very limited expertise in isotemporal substitution models and therefore cannot comment on this aspect of the manuscript.

My main concern revolves around the estimates of the SB classifier used for this study. The median total sedentary behaviour (SB) duration for this analysis (12.3 hours per day, see Table 1) seems fairly high compared to other studies that have applied machine learning classifiers to UK Biobank data. For instance, the widely used RF+HMM classifier by Walmsley et al. (2022) yields a median SB duration of 9.2-9.3 hours per day (see Le et al., 2025, or the original Walmsley study). Results from thigh-worn accelerometer studies (e.g. Ahmadi et al., 2024), which offer greater accuracy in measuring free-living SB, show SB ranging from 9.0 to 10.3 hours per day. Overall, this makes me wonder about the amounts of SB classified here.

Matthews et al. (2025) very recently published a detailed comparison of machine learning and cut-point-based approaches for classifying SB using wrist-worn accelerometers. It would be helpful if the authors could elaborate further on the validity of their ML classifier in light of these findings.

References:

Ahmadi, M. N., Blodgett, J. M., Atkin, A. J., Chan, H.-W., Del Pozo Cruz, B., Suorsa, K., Bakker, E. A., Pulsford, R. M., Mielke, G. I., Johansson, P. J., Hettiarachchi, P., Thijssen, D. H. J., Stenholm, S., Mishra, G. D., Teixeira-Pinot, A., Rangul, V., Sherar, L. B., Ekelund, U., Hughes, A. D., … Stamatakis, E. (2024). Relationship of device measured physical activity type and posture with cardiometabolic health markers: Pooled dose-response associations from the Prospective Physical Activity, Sitting and Sleep Consortium. Diabetologia, 67(6), 1051-1065. [https://eur03.safelinks.protection.outlook.com/?url=https%3A%2F%2Fdoi.org%2F10.1007%2Fs00125-024-06090-y&data=05%7C02%7CFrederick.Ho%40glasgow.ac.uk%7Cbd29478a25254ae463ec08de397055c7%7C6e725c29763a4f5081f22e254f0133c8%7C1%7C0%7C639011351830679410%7CUnknown%7CTWFpbGZsb3d8eyJFbXB0eU1hcGkiOnRydWUsIlYiOiIwLjAuMDAwMCIsIlAiOiJXaW4zMiIsIkFOIjoiTWFpbCIsIldUIjoyfQ%3D%3D%7C0%7C%7C%7C&sdata=uD0eQDTd86MMv%2B7r%2B%2F9lG2uH0KXKeSgUvbGMzVEVdJQ%3D&reserved=0](https://eur03.safelinks.protection.outlook.com/?url=https%3A%2F%2Fdoi.org%2F10.1007%2Fs00125-024-06090-y&data=05%7C02%7C2431055Z%40student.gla.ac.uk%7C172465965c1e4c01c49a08de3970c7c9%7C6e725c29763a4f5081f22e254f0133c8%7C1%7C0%7C639011353705744415%7CUnknown%7CTWFpbGZsb3d8eyJFbXB0eU1hcGkiOnRydWUsIlYiOiIwLjAuMDAwMCIsIlAiOiJXaW4zMiIsIkFOIjoiTWFpbCIsIldUIjoyfQ%3D%3D%7C0%7C%7C%7C&sdata=th7VQXX7Q59B8JEyXjUd3tGP7EoX%2BvoovtiIIe2DzLw%3D&reserved=0)

Le, F., Dumuid, D., D'Elia, O., Haussmann, A., Stamatakis, E., Doherty, A., Liao, Y., & Wiley, J. F. (2025). 24-hour Physical Activity, Sedentary, and Sleep Profiles in Individuals with Cancer: A UK Biobank Cohort Study. medRxiv. [https://eur03.safelinks.protection.outlook.com/?url=https%3A%2F%2Fdoi.org%2F10.1101%2F2025.02.25.25322841&data=05%7C02%7CFrederick.Ho%40glasgow.ac.uk%7Cbd29478a25254ae463ec08de397055c7%7C6e725c29763a4f5081f22e254f0133c8%7C1%7C0%7C639011351830696121%7CUnknown%7CTWFpbGZsb3d8eyJFbXB0eU1hcGkiOnRydWUsIlYiOiIwLjAuMDAwMCIsIlAiOiJXaW4zMiIsIkFOIjoiTWFpbCIsIldUIjoyfQ%3D%3D%7C0%7C%7C%7C&sdata=qbxTByXJ033UKPnXyYm9yz1eb3Ll%2BU%2FmN3ASqpcnGEY%3D&reserved=0](https://eur03.safelinks.protection.outlook.com/?url=https%3A%2F%2Fdoi.org%2F10.1101%2F2025.02.25.25322841&data=05%7C02%7C2431055Z%40student.gla.ac.uk%7C172465965c1e4c01c49a08de3970c7c9%7C6e725c29763a4f5081f22e254f0133c8%7C1%7C0%7C639011353705757948%7CUnknown%7CTWFpbGZsb3d8eyJFbXB0eU1hcGkiOnRydWUsIlYiOiIwLjAuMDAwMCIsIlAiOiJXaW4zMiIsIkFOIjoiTWFpbCIsIldUIjoyfQ%3D%3D%7C0%7C%7C%7C&sdata=%2BvzsWSu4KcbgqRULztEHzQjRjGpncC3H08hjyilOY9E%3D&reserved=0)

Matthews, C. E., Saint-Maurice, P., Freeman, J. R., Hayes, H. A., Shreves, A. H., Doherty, A., Hyde, E. T., Ylarregui, K., Jones, R. R., & Keadle, S. K. (2025). Performance evaluation of algorithms to estimate daily sedentary time using wrist-worn sensors in free-living adults. Journal for the measurement of physical behaviour, 8(1), jmpb.2024-0051. [https://eur03.safelinks.protection.outlook.com/?url=https%3A%2F%2Fdoi.org%2F10.1123%2Fjmpb.2024-0051&data=05%7C02%7CFrederick.Ho%40glasgow.ac.uk%7Cbd29478a25254ae463ec08de397055c7%7C6e725c29763a4f5081f22e254f0133c8%7C1%7C0%7C639011351830712203%7CUnknown%7CTWFpbGZsb3d8eyJFbXB0eU1hcGkiOnRydWUsIlYiOiIwLjAuMDAwMCIsIlAiOiJXaW4zMiIsIkFOIjoiTWFpbCIsIldUIjoyfQ%3D%3D%7C0%7C%7C%7C&sdata=V9uYyIGmOM8r9JU88yg8RYuo%2FYcU%2BNj9IBHHvNAaCiI%3D&reserved=0](https://eur03.safelinks.protection.outlook.com/?url=https%3A%2F%2Fdoi.org%2F10.1123%2Fjmpb.2024-0051&data=05%7C02%7C2431055Z%40student.gla.ac.uk%7C172465965c1e4c01c49a08de3970c7c9%7C6e725c29763a4f5081f22e254f0133c8%7C1%7C0%7C639011353705771621%7CUnknown%7CTWFpbGZsb3d8eyJFbXB0eU1hcGkiOnRydWUsIlYiOiIwLjAuMDAwMCIsIlAiOiJXaW4zMiIsIkFOIjoiTWFpbCIsIldUIjoyfQ%3D%3D%7C0%7C%7C%7C&sdata=%2B1C%2F8OOafPaOmVowEQhy65JFiwX4fwJQo5r0oTBQp6E%3D&reserved=0)

Walmsley, R., Chan, S., Smith-Byrne, K., Ramakrishnan, R., Woodward, M., Rahimi, K., Dwyer, T., Bennett, D., & Doherty, A. (2022). Reallocation of time between device-measured movement behaviours and risk of incident cardiovascular disease. British Journal of Sports Medicine, 56(18), 1008-1017.

RESPONSE: Thank you very much for pointing our the inconsistency between the SB values and the published data. The original scoring of the SB was based on earlier version of programming codes supplied by an author Trost. Upon careful review, we found that the codes used a customized rule in separating non-wear from sleep. However, we have not revised the codes to conform with the standard GGIR rule in determining non-wear and sleep. The resulting numbers are close to what have been published. It appears the difference was non-differential and none of the conclusions from the original analyses changed.

Specific comments:

Abstract
- Page 2: 'A two-step approach based on a random forest model was used to classify wear time.' This sentence reads awkwardly, as if the goal was to classify wear time rather than SB. I suggest rephrasing it as follows: 'A two-step approach based on a random forest model was used to classify SB based on features calculated from raw acceleration.'

RESPONSE: Thank you for your suggestion, the sentence now has been changes to your comments.

Methods
- Page 6: Could the authors please provide more detail on their exclusion criteria for the accelerometer data? What threshold was used to define poor device calibration? Was it any 72 hours within the seven-day period, or three full days, for example?

RESPONSE: In the rescoring the accelerometer data, we have tightened the inclusion criteria to have at least 4 days (96 hours) of valid (i.e. excluding non-wear) wear time. However, the <3 days (<72 hours) of non-wear time can be distributed across different time. The exclusion was done after the scoring which classified non-wear based on GGIR rule. These have been added to the paper.

- Page 7: It remains unclear to me which ML model was actually used. As Matthews et al. (2025) report, there seem to be multiple variations of the RF model underlying the work of Pavey et al. (2018). Could the authors please elaborate on the naming convention used by Matthews et al. and specify which classifier was actually used (i.e. Trost Original/Extended)? Furthermore, I would suggest explicitly stating the classification performance metrics for SB during previous validations (i.e. recall/precision). The authors state that the model has been published before. Is the model itself openly accessible, for example via GitHub or an R/Python package such as 'actimetric'? If so, it would be helpful to provide a direct reference here to avoid confusion.

RESPONSE: We used the ‘Adult Wrist RF Trost’ algorithm in the now publicly available actimetric package (<https://github.com/PhysicalActivityOpenTools/actimetric>) where Matthews and Trost are co-developers. The accuracy metrics (PPV and NPV) are also added.

- Page 7: The authors define prolonged and intermittent bouts of physical activity, but these do not seem to be used for anything within the analysis. If I am not mistaken, these definitions are irrelevant to this paper and could be removed to avoid confusing the reader. Additionally, it would be beneficial to explicitly define interrupted SB in addition to prolonged SB, and to state that these two types of SB are mutually exclusive.

RESPONSE: Indeed bouted PA were not used in this paper and the description has been removed. We have added the definition of interrupted SB and that the 2 types of SBs are mutually exclusive.

- Page 12: Shouldn't it read 'Coefficients β1 to β4' instead? Otherwise, the authors may need to rephrase the sentence, i.e. by adding type-specific SB for the fifth coefficient.

RESPONSE: We have revised the formula to be more explicit two different ISM were used. We did not report or interpret the coefficient for sleep because sleep duration is more likely to be confounded by mental health and outside the scope of the study.

Results

- Page 13: There seems to be a typo 'Table 2 shows the associations between associations between a 1-hour increment in overall [...]'.

RESPONSE: The typo of Table 2 now has been corrected.

Discussion

- Page 19: It would be great to see a discussion about the possibility of SB being overestimated and how this could potentially influence the presented findings.

RESPONSE: With the rescoring of the data it does not appear the risk of overestimation to be high.

Supplementary

\# Supplementary Table 2: There seems to be an error for the duration of Q4. It currently says '> 24.00' which is likely incorrect and should probably read '> 7.62'.

RESPONSE: Supplementary Table 2 now has been corrected.

\# Supplementary Table 3: There is a similiar issue with Q4 (see my comment on Suppl. Table 2).

RESPONSE: Thank you. Supplementary Table 3 now has been corrected

Reviewer #3: This is a well-written and interesting manuscript reporting on the associations between prolonged and interrupted sedentary behavior and cancer-related mortality outcomes. Minor comments are noted below for the authors' consideration:

1. Please include information as to the average length of follow-up in the sample to better contextualize the results.

RESPONSE: Thank you for your comments. We have revised the manuscript to include follow-up duration for the sample. The median follow-up time was 12.38 years (IQR, 11.56–13.15 years), which has now been added to the Methods section.

2. To the above point, did the authors consider the possibility of confounding by health status (see PMC7669589)? This is a worthwhile consideration in accelerometry-mortality analyses, particularly if the length of follow-up time is <6 years.

RESPONSE: We agree that there is a risk that preexisting health conditions could confound the relationship and have therefore included the long-term condition variable as a mitigation. However, it is also possible these variables are mediators as sedentary behaviors could cause some of the long-term conditions. We have added a discussion on this in the Limitations.

3. Can the authors explain why they chose to explore the association between sedentary behavior and cancer-related mortality outcomes in a sample of cancer-free adults, rather than a cancer population? Did the authors also explore all-cause mortality?

RESPONSE: Our objective was to evaluate sedentary behavior as a pre-diagnostic exposure in relation to subsequent cancer incidence and cancer mortality among adults who were cancer-free at baseline. A cohort restricted to individuals with cancer would address a different question, namely prognosis or survival after diagnosis. We have clarified this rationale in the Discussion. Further complicate the analysis for cancer survival is that cancer diagnosis change behavior and the assessment of sedentary behavior need to be timed accurately, with repeated measures to unpack the nuances. Unfortunately we do not have such data in UK Biobank. We did not examine all-cause mortality in the present study, as our primary focus was cancer-specific outcomes.

4. To the above point, are the authors able to comment as to whether PA and SB behaviors were measured prior to or after a diagnosis of cancer, which preceded the mortality outcome? This is an important consideration when messaging about lifestyle behavior change.

RESPONSE: Movement behaviors were measured during the baseline accelerometer assessment in participants who were free of cancer at study entry. Therefore, for those who later died from cancer, PA and SB were measured before the diagnosis leading to the mortality outcome. We have clarified this temporal ordering in the manuscript and now explicitly state that our results pertain to pre-diagnostic behaviors and should not be interpreted as direct evidence about post-diagnostic lifestyle modification among patients with cancer.
