# Supplementary material for: Accelerometry-measured prolonged and interrupted sedentary behavior and cancer incidence and mortality: A cohort study of 91,292 UK Biobank participants
Source: PLoS Med. 2026 Jul 2;23(7):e1004767. doi: 10.1371/journal.pmed.1004767 (PMC13327276; doi:10.1371/journal.pmed.1004767)
Supplement: Attachment — Submitted filename: minor revision_response letter.docx [file pmed.1004767.s004.docx]

Requests from Editors:

GENERAL EDITORIAL REQUESTS

* Please confirm that your title complies with PLOS Medicine's style. Your title must be nondeclarative and not a question. It should begin with main concept if possible. "Effect of" should be used only if causality can be inferred, i.e., for an RCT. Please place the study design ("A randomized controlled trial," "A retrospective study," "A modelling study," etc.) in the subtitle (ie, after a colon).

* Please confirm that your abstract complies with our requirements, including format (three sections: Background, Methods and Findings, and Conclusions) and providing all the information relevant to this study type <https://journals.plos.org/plosmedicine/s/submission-guidelines#loc-abstract>

The abstract with three sections followed the journal requirements format.

* In the abstract, please include the important dependent variables that are adjusted for in the analyses.

The dependent variables that were adjusted for the analyses now has been added in the Abstract.

* In the last sentence of the Abstract Methods and Findings section, please describe the main limitation(s) of the study's methodology.

The main limitations of the study’s methodology now have been added in the Abstract Methods and Findings section.

* In the author summary, in the final bullet point of 'What Do These Findings Mean?', please include the main limitations of the study in non-technical language. Please see our author guidelines for more information: <https://journals.plos.org/plosmedicine/s/revising-your-manuscript#loc-author-summary>."

The author summary now has been following the requirements of three bullet points. The main limitations of the study have been added in the final bullet point in non-technical language.

* Please ensure that the Introduction ends with a clear description of the study question or hypothesis.

The Introduction ends now has been rewrite with study hypothesis.

* Statistical reporting: Please revise throughout the manuscript, including tables and figures.

- Please report statistical information as follows to improve clarity for the reader 22% (95% CI [13,28]; p</=).
- Please separate upper and lower bounds with commas instead of hyphens as the latter can be confused with reporting of negative values.
- Please repeat statistical definitions (HR, CI etc.) for each set of parentheses.

Edited both manuscript and tables follow the above requirements.

* Please remove the 'conclusions' subheading from the discussion. Please also remove any other subheadings from the discussion.

All subheadings from the discussion now have been removed

* Please ensure that all abbreviations are defined at first use throughout the text.

Thank you. All abbreviations were defined at first use throughout the manuscript.

* Please confirm that all numbers presented in the abstract are present and identical to numbers presented in the main manuscript text.

All numbers now consist of the manuscript.

FUNDING STATEMENT

* The funding statement should include specific grant numbers, initials of authors who received each award, URLs to sponsors’ websites. Also, please state whether any sponsors or funders (other than the named authors) played any role in study design, data collection and analysis, the decision to publish, or preparation of the manuscript. If they had no role in the research, include this sentence: “The funders had no role in study design, data collection and analysis, decision to publish, or preparation of the manuscript.”

Edited and updated the funding statement follow the requirements above.

COMPETING INTERESTS STATEMENT

* All authors must declare their relevant competing interests per the PLOS policy, which can be seen here: https://journals.plos.org/plosmedicine/s/competing-interests For authors with ties to industry, please indicate whether any of the interests has a financial stake in the results of the current study.

* Please add this statement to the manuscript's Competing Interests: "[Initials] is a paid statistical consultant on PLOS Medicine's statistical board."

The statement of ‘FH is a paid statistical consultant on PLOS Medicine's statistical board’ now has been added to the Competing interests. The rest authors no relevant competing interests declare.

DATA AVAILABILITY

* PLOS defines the “minimal data set” to consist of the data set used to reach the conclusions drawn in the manuscript with related metadata and methods, and any additional data required to replicate the reported study findings in their entirety. Authors do not need to submit their entire data set, or the raw data collected during an investigation. Please submit the following data:

The values behind the means, standard deviations and other measures reported;
The values used to build graphs;
The points extracted from images for analysis.

* The Data Availability Statement (DAS) requires revision. If the data are not freely available, please describe briefly the ethical, legal, or contractual restriction that prevents you from sharing it. Please also include an appropriate contact (web or email address) for inquiries (again, this cannot be a study author).

The Data Availability now has been edited follow the requirements.

ETHICS AND CONSENT

* Please move the following sentence from the acknowledgements ethics section and clarify in the statement f the numbers provided refer to approval for data use: 'This research has been conducted using the UK Biobank Resource under Application Number 7155 and 46466.'

The sentence has been moved from the Acknowledgement, and the Ethics and Consent has been edited follow the requirement.

FIGURES

* Please consider moving the flow diagram shared in the supplementary information as Figure 1 into the main text to aid the reader.

The flow diagram now has been moved to Figure 1 into the main text.

* Please provide titles and legends for all figures and tables (including those in Supporting Information files). Please define all acronyms used in each figure or table in its corresponding legend.

The titles and legends for all figures and tables now have been edited according to the requirement.

* Please ensure that where relevant figures include 95% CIs.

95% CI included in the relevant figures.

* Please consider avoiding the use of green in order to make your figure more accessible

The figures now have been changed color from green to purple

* Please provide the unadjusted comparisons as well as the adjusted comparisons in all relevant Tables

The unadjusted outcomes by sedentary behavior categories are shown in the descriptive tables (e.g. Table 1 for total sedentary behavior).

* Please specify the variables controlled for in all relevant Tables

All adjusted variables were mentioned in the with the footnote of Tables.

OBSERVATIONAL, COHORT, CROSS-SECTIONAL, AND CASE CONTROL STUDIES

* Please ensure that the study is reported according to the STROBE guideline and include the completed STROBE checklist as Supporting Information. Please add the following statement, or similar, to the Methods: ""This study is reported as per the Strengthening the Reporting of Observational Studies in Epidemiology (STROBE) guideline (S1 Checklist).""

The statement has been added to the Methods, line 245-246.

The STROBE guideline can be found here: http://www.equator-network.org/reporting-guidelines/strobe/

When completing the checklist, please use section and paragraph numbers, rather than page numbers.

* Did your study have a prospective protocol or analysis plan? Please state this (either way) early in the Methods section.

a) If a prospective analysis plan (from your funding proposal, IRB or other ethics committee submission, study protocol, or other planning document written before analyzing the data) was used in designing the study, please include the relevant prospectively written document with your revised manuscript as a Supporting Information file to be published alongside your study, and cite it in the Methods section. A legend for this file should be included at the end of your manuscript.

b) If no such document exists, please make sure that the Methods section transparently describes when analyses were planned, and when/why any data-driven changes to analyses took place.

c) In either case, changes in the analysis-- including those made in response to peer review comments-- should be identified as such in the Methods section of the paper, with rationale."
* Your study is observational and therefore causality cannot be inferred. Please remove language that implies causality and refer to associations instead.

We have added this statement in the statistical analysis section: There was no prospective analysis protocol for this study and there were no changes to the analysis plan. The data was re-analyzed once during the peer review process due to a significant update to the actimetry package used to ascertain SB and PA.

* For all observational studies, in the manuscript text, please indicate: (1) the specific hypotheses you intended to test, (2) the analytical methods by which you planned to test them, (3) the analyses you actually performed, and (4) when reported analyses differ from those that were planned, transparent explanations for differences that affect the reliability of the study's results. If a reported analysis was performed based on an interesting but unanticipated pattern in the data, please be clear that the analysis was data driven.

We have added hypotheses to the end of the Introduction, and the remaining were reported in the Methods – Statistical analysis section.

Comments from Reviewers:

Reviewer #1: All my comments have been addressed


Reviewer #3: The authors were comprehensive in their responses to the reviewers and sufficiently addressed the comments. The resulting manuscript has been strengthened by the revisions.


Any attachments provided with reviews can be seen via the following link:
********

Journal Requirements:

1) [ STROBE_checklist_cohort (1).docx] is currently uploaded as file type “Other”, which is not viewable by the reviewers. Please change the file type(s) to 'Supporting Information' and include a legend in the manuscript if you wish it/them to be included in review.

Thank you for your reminder. The checklist file will upload as file type ‘Supporting information’ and the legend now has been in the manuscript.

2) We note that your Supporting Information files are duplicated on your submission. Please remove any unnecessary or old files from your revision and make sure that only those relevant to the current version of the manuscript are included.

The duplicated and unnecessary files now have been removed. The supporting information files now included: supplementary materials, supplementary figure and STROBE checklist.
