# Supplementary material for: Accelerometry-measured prolonged and interrupted sedentary behavior and cancer incidence and mortality: A cohort study of 91,292 UK Biobank participants
Source: PLoS Med. 2026 Jul 2;23(7):e1004767. doi: 10.1371/journal.pmed.1004767 (PMC13327276; doi:10.1371/journal.pmed.1004767)
Supplement: Peer review history

# Editor report: Decision Letter 0

**DOI:** 10.1371/journal.pmed.1004767.r001

**Authors:** Jennifer Thorley

16 Sep 2025

Dear Dr Ho,

Thank you for submitting your manuscript entitled "Accelerometry-measured prolonged and interrupted sedentary behavior and cancer incidence and mortality: A prospective cohort study of 92,354 UK Biobank participants" for consideration by PLOS Medicine.

Your manuscript has now been evaluated by the PLOS Medicine editorial staff as well as by an academic editor with relevant expertise and I am writing to let you know that we would like to send your submission out for external peer review.

However, before we can send your manuscript to reviewers, we need you to complete your submission by providing the metadata that is required for full assessment. To this end, please login to Editorial Manager where you will find the paper in the 'Submissions Needing Revisions' folder on your homepage. Please click 'Revise Submission' from the Action Links and complete all additional questions in the submission questionnaire.

For clinical studies, please upload a copy of your trial study protocol as a supporting information file. The study protocol should be the version submitted for approval to the institutional review board or ethics committee, should include any amendments to the study protocol, as well as the date of their approval by the institutional review or ethics committee. Please also detail any deviations from the study protocol in the Methods section of your manuscript. The editors will consider the protocol and study conduct prior to a final decision for external review.

Please re-submit your manuscript within two working days, i.e. by Sep 18 2025 11:59PM.

Login to Editorial Manager here: https://www.editorialmanager.com/pmedicine

Once your full submission is complete, your paper will undergo a series of checks in preparation for peer review. Once your manuscript has passed all checks it will be sent out for review.

Feel free to email us at plosmedicine@plos.org if you have any queries relating to your submission.

Kind regards,

Jennifer Thorley

PLOS Medicine

# Peer review: Decision Letter 1

**DOI:** 10.1371/journal.pmed.1004767.r002

**Authors:** Jennifer Thorley, Andreia Cunha

12 Dec 2025

Dear Dr Ho,

Apologies for the delay in getting back to you with a decision, which was due to challenges in securing all the necessary advice. Many thanks for submitting your manuscript "Accelerometry-measured prolonged and interrupted sedentary behavior and cancer incidence and mortality: A prospective cohort study of 92,354 UK Biobank participants" (PMEDICINE-D-25-03223R1) to PLOS Medicine. The paper has been reviewed by subject experts and a statistician; their comments are included below and can also be accessed here: [LINK]

As you will see, the reviewers find your work of potential interest, but they raise some important questions and concerns that we must ask you to address in full. After discussing the paper with the editorial team and an academic editor with relevant expertise, I'm pleased to invite you to revise the paper in response to the reviewers' comments. We plan to send the revised paper to some or all of the original reviewers, and we cannot provide any guarantees at this stage regarding publication.

In addition to these revisions, you may need to complete some formatting changes, which you will receive in a follow up email. A member of our team will be in touch with a set of requests shortly. If you do not receive a separate email within a few days, please assume that checks have been completed, and no additional changes are required.

When you upload your revision, please include a point-by-point response that addresses all of the reviewer and editorial points, indicating the changes made in the manuscript and either an excerpt of the revised text or the location (eg: page and line number) where each change can be found. Please also be sure to check the general editorial comments at the end of this letter and include these in your point-by-point response. When you resubmit your paper, please include a clean version of the paper as the main article file and a version with changes tracked as a marked-up manuscript. It may also be helpful to check the guidelines for revised papers at http://journals.plos.org/plosmedicine/s/revising-your-manuscript for any that apply to your paper.

We ask that you submit your revision by Jan 30 2026 11:59PM. However, if this deadline is not feasible, please contact me by email, and we can discuss a suitable alternative.

Don't hesitate to contact me directly with any questions (acunha@plos.org).

Best regards,

Andreia

Andreia Cunha, PhD

Senior editor

PLOS Medicine

acunha@plos.org

-----------------------------------------------------------

Comments from the academic editor:

Accelerometry-measured prolonged and interrupted sedentary behavior and cancer incidence and mortality: A prospective cohort study of 92,354 UK Biobank participants

This manuscript examines the association of interrupted for prolonged sedentary behaviors (SB) on risk of incident cancer and cancer mortality. Through isotemporal models, the authors show that prolonged and interrupted SB have diverging associations with risk, with prolonged SB associated with increased risk and interrupted SB associated with decreased risk, all while holding levels of other activity constant. This provides an important proof of concept for the SB construct—it’s not just your total physical activity level, but whether your activity is used to break up bouts of long sitting. That’s a distinct scientific contribution that sets the paper apart from others in the literature.

In general, the analysis is well conducted and thoughtful, getting at key concepts more directly than prior papers. The presentation of the methods and results for the isotemporal models still need work, though, as the paper is sometimes hard to follow. I have several suggestions that may help improve readability and strengthen the science.

Major comments

1. “Replacing with” and “substituting with”: The manuscript uses these two phrases interchangeably. While this is grammatically acceptable, “substituting X with Y” is inherently ambiguous: most readers will interpret it correctly as “replacing X with Y,” but a nontrivial minority may read it as “adding X in place of Y.” Using two different phrases to express the same idea compounds this ambiguity. In contrast, “replacing with” is unambiguous. I recommend eliminating “substituting with” and using “replacing with” consistently throughout for greater clarity.

2. Methods and isotemporal substitution model (ISMs): The analysis examined two distinct ISMs. The authors should present each individually in the methods and define the meaning of terms specific to each model separately. This will substantially clarify the text on B1 to B5, which is presently incomplete (no description of B5) and logically incorrect (the effects describe replacing a specific kind of SB, not just any SB).

3. Editing: The quality of editing declines toward the end of the manuscript, with several copy errors, some awkward phrasings, a few incomplete thoughts, and occasional non sequiturs. Given the complexity of the models, the added uncertainty about the text’s accuracy substantially increases the cognitive burden on readers. The authors should check the document carefully.

Some phrases that need fixing:

“Table 2 shows the associations between associations between..” (p.13)

“both total sedentary time and sedentary bout duration…it had” (p.16—what is “it”?)

“which consist of the results of overall cancer incident with our study” (p.17)

“which still partly had the similar individual results as our study” (p.17)

“The ISM results from this study consistent with” (p.17)

“Additionally, the dose-response association for risk of cancer mortality when total sedentary time and mean sedentary bout length were expressed prolongedly” (p.17)

“This sheds light on the mechanism how SB could influence health outcomes – both by itself accumulated in a prolonged manner, as well as to replace PA.” (p.17-18—I’m not sure what the point is here. Authors should make the sentence direct and punchy—how exactly does it shed light?)

“In particular, it has been shown that device measured total SB was associated with endothelial dysfunction and lowgrade inflammation, adjusted for PA (35). (p.18—is this relevant given this paper’s focus on “total SB”? Especially considering the next sentence..)

“But no significant association with sedentary breaks or average length of sedentary bouts, potentially due to the different operationalization of interrupting SB, as well as their smaller sample size (n=2363).” (p.18—the sentence lacks a verb/predicate)

“even when body fat is not a factor” (p.18—this is immediately followed by a sentence about why fat is important. Perhaps this meant to say body fat percent? Regardless, that’s not the entry point I would use for this topic)

“A particular concern is the accumulation of ectopic fat…” (p.18—how specifically is this a concern with respect to SB-cancer associations? Are SBs associated with ectopic fat? The passively constructed argument here makes it hard to tell whether there is a logically complete argument).

“Insulin and insulin-like growth factor I (IGF-I) are anabolic hormones that play critical roles in regulating glucose metabolism, cell growth, apoptosis, and angiogenesis (32). Excessive activation of these hormones, along with their associated binding proteins (IGFBP-1 through -6) and signaling pathways, has been linked to an increased risk of several cancers, including breast, prostate, and colorectal cancer (32).” (p.18—Are these factors linked with SB? To what extent? Specifically with prolonged sitting?)

“Second, it used validated, machine learning-informed, approach to derive the SB which is more accurate than the questionnaire-based study” (p.19)

“which has been shown using a different analysis” (p.20—as phrased, this will make the reader think the point is the use of a different model, not the use of the same study population. Authors should amend).

4. Comorbidities: The lack of adjustment for comorbidities, e.g. diabetes, will be a major concern for many readers. I appreciate the sensitivity analysis (and the point about “confounders or mediators”) but the methods and results should include more details about this analysis, since it is rather important. Authors should describe the construction of the comorbidity count in the main text (including the list of comorbidities considered and any weighting) and provide more details on the results.

Minor comments

5. Abstract: In the methods, covariate adjustments should be noted. In the results, authors should aim for parallel construction of the last two sentences, and use the correct subscripts after HR.

6. Introduction: Authors should briefly describe the biological rationale. I recall interventional studies of volunteers with prolonged sitting that examined levels of several biomarkers (e.g. Dunstan et al., 2012; Peddie et al., 2013). Perhaps these are relevant?

7. Cancer endpoints: Esophageal—please clarify the percent that are esophageal adenocarcinoma. Multiple myeloma—this is a general code for plasma cell neoplasms (most of which are multiple myeloma). I’m fine with using this code, but a minor clarification is in order. Leukemia—I’m fine with this coding, but studies increasingly split this by cell lineage, i.e. myeloid or lymphoid. Multiple melanoma—delete the word multiple here.

8. Discussion: Consistent with the concerns outlined in the “Editing” section above, the Mechanisms section should be revised extensively to improve clarity.

9. Tables and figures: Table 1: Values flipped for sugar intake in the total column. Smoking should be added, also alcohol. All items should be listed in the same order as Footnote to Table 2 (with variables for sensitivity analysis last, as currently done). Table 4. The “ratio of HR” is space-efficient, but most readers will misunderstand it. I would either present the full set of results or just use the p-values for interaction. The title needs to indicate whether this is the ISM or non-ISM model. If the latter, please include before any ISM models are presented. The word “obesity” should be replaced by “adiposity” (obesity is specific to BMI).

-----------------------------------------------------------

Comments from the reviewers:

Reviewer #1: This manuscript describes results from an analysis of the UK biobank on the association between interrupted and prolonged SB on cancer. My comments are:

- A Lot of the hazard ratios are rather small and are significant mostly as a result of a large sample size and therefore the concept of clinically significant becomes more important. Are these hazard ratios clinically significant?

- "The optimal strategy for replacing prolonged sedentary behavior will vary based on individual ability and preferences." Is that something that your analysis shows?

- "Participants with insufficient wear time (<72 hours), missing data, or poor device calibration were excluded." How many were these, especially the missing data part? Complete case analysis is not recommended unless the amount of missing data is very low and methods such as imputation should be used.

- Is the variable Prolonged PA measured once or is it time-dependent?

- "Coefficients β₁ to β₅ represented the effects of substituting SB with sleep, LPA, MPA, and VPA respectively, while holding total time constant." I have a hard time understanding how the "intervention" variable was defined. Is it a categorical variable with the levels in the equation on page 11. I assume people can satisfy more than one of these levels. How were they classified? More generally, I think a better description of the intervention would be helpful.

- "Table 3 shows the risks of composite cancer outcomes when one type of SB was replaced by the other type of SB, LPA, MPA, or VPA." I don't think this is the correct interpretation of hazard ratios and risk and hazard ratios seem to be used to mean the same thing throughout, which is not exactly correct either.

Reviewer #2: Dear authors,

Thank you for giving me the opportunity to review this interesting manuscript.

In their study, the authors investigated the cancer risk associated with prolonged and interrupted sedentary behaviour. They used prospective data from the UK Biobank and a random forest classifier to quantify sedentary behaviour from wrist-worn accelerometer data. The manuscript provides a thorough analysis, including a detailed sensitivity analysis. The findings of this study advance our understanding of the detrimental effects of uninterrupted sedentary behaviour compared to breaking up sitting. The manuscript is well written and follows a clear structure.

I have a few comments which I hope will help the authors further improve it. I must acknowledge, however, that I have very limited expertise in isotemporal substitution models and therefore cannot comment on this aspect of the manuscript.

My main concern revolves around the estimates of the SB classifier used for this study. The median total sedentary behaviour (SB) duration for this analysis (12.3 hours per day, see Table 1) seems fairly high compared to other studies that have applied machine learning classifiers to UK Biobank data. For instance, the widely used RF+HMM classifier by Walmsley et al. (2022) yields a median SB duration of 9.2-9.3 hours per day (see Le et al., 2025, or the original Walmsley study). Results from thigh-worn accelerometer studies (e.g. Ahmadi et al., 2024), which offer greater accuracy in measuring free-living SB, show SB ranging from 9.0 to 10.3 hours per day. Overall, this makes me wonder about the amounts of SB classified here.

Matthews et al. (2025) very recently published a detailed comparison of machine learning and cut-point-based approaches for classifying SB using wrist-worn accelerometers. It would be helpful if the authors could elaborate further on the validity of their ML classifier in light of these findings.

References:

Ahmadi, M. N., Blodgett, J. M., Atkin, A. J., Chan, H.-W., Del Pozo Cruz, B., Suorsa, K., Bakker, E. A., Pulsford, R. M., Mielke, G. I., Johansson, P. J., Hettiarachchi, P., Thijssen, D. H. J., Stenholm, S., Mishra, G. D., Teixeira-Pinot, A., Rangul, V., Sherar, L. B., Ekelund, U., Hughes, A. D., … Stamatakis, E. (2024). Relationship of device measured physical activity type and posture with cardiometabolic health markers: Pooled dose-response associations from the Prospective Physical Activity, Sitting and Sleep Consortium. Diabetologia, 67(6), 1051-1065. https://doi.org/10.1007/s00125-024-06090-y

Le, F., Dumuid, D., D'Elia, O., Haussmann, A., Stamatakis, E., Doherty, A., Liao, Y., & Wiley, J. F. (2025). 24-hour Physical Activity, Sedentary, and Sleep Profiles in Individuals with Cancer: A UK Biobank Cohort Study. medRxiv. https://doi.org/10.1101/2025.02.25.25322841

Matthews, C. E., Saint-Maurice, P., Freeman, J. R., Hayes, H. A., Shreves, A. H., Doherty, A., Hyde, E. T., Ylarregui, K., Jones, R. R., & Keadle, S. K. (2025). Performance evaluation of algorithms to estimate daily sedentary time using wrist-worn sensors in free-living adults. Journal for the measurement of physical behaviour, 8(1), jmpb.2024-0051. https://doi.org/10.1123/jmpb.2024-0051

Walmsley, R., Chan, S., Smith-Byrne, K., Ramakrishnan, R., Woodward, M., Rahimi, K., Dwyer, T., Bennett, D., & Doherty, A. (2022). Reallocation of time between device-measured movement behaviours and risk of incident cardiovascular disease. British Journal of Sports Medicine, 56(18), 1008-1017.

Specific comments:

_# Abstract

- Page 2: 'A two-step approach based on a random forest model was used to classify wear time.' This sentence reads awkwardly, as if the goal was to classify wear time rather than SB. I suggest rephrasing it as follows: 'A two-step approach based on a random forest model was used to classify SB based on features calculated from raw acceleration.'

_# Methods

- Page 6: Could the authors please provide more detail on their exclusion criteria for the accelerometer data? What threshold was used to define poor device calibration? Was it any 72 hours within the seven-day period, or three full days, for example?

- Page 7: It remains unclear to me which ML model was actually used. As Matthews et al. (2025) report, there seem to be multiple variations of the RF model underlying the work of Pavey et al. (2018). Could the authors please elaborate on the naming convention used by Matthews et al. and specify which classifier was actually used (i.e. Trost Original/Extended)? Furthermore, I would suggest explicitly stating the classification performance metrics for SB during previous validations (i.e. recall/precision). The authors state that the model has been published before. Is the model itself openly accessible, for example via GitHub or an R/Python package such as 'actimetric'? If so, it would be helpful to provide a direct reference here to avoid confusion.

- Page 7: The authors define prolonged and intermittent bouts of physical activity, but these do not seem to be used for anything within the analysis. If I am not mistaken, these definitions are irrelevant to this paper and could be removed to avoid confusing the reader. Additionally, it would be beneficial to explicitly define interrupted SB in addition to prolonged SB, and to state that these two types of SB are mutually exclusive.

- Page 12: Shouldn't it read 'Coefficients β1 to β4' instead? Otherwise, the authors may need to rephrase the sentence, i.e. by adding type-specific SB for the fifth coefficient.

_# Results

- Page 13: There seems to be a typo 'Table 2 shows the associations between associations between a 1-hour increment in overall [...]'.

_# Discussion

- Page 19: It would be great to see a discussion about the possibility of SB being overestimated and how this could potentially influence the presented findings.

_# Supplementary

# Supplementary Table 2: There seems to be an error for the duration of Q4. It currently says '> 24.00' which is likely incorrect and should probably read '> 7.62'.

# Supplementary Table 3: There is a similiar issue with Q4 (see my comment on Suppl. Table 2).

Reviewer #3: This is a well-written and interesting manuscript reporting on the associations between prolonged and interrupted sedentary behavior and cancer-related mortality outcomes. Minor comments are noted below for the authors' consideration:

1. Please include information as to the average length of follow-up in the sample to better contextualize the results.

2. To the above point, did the authors consider the possibility of confounding by health status (see PMC7669589)? This is a worthwhile consideration in accelerometry-mortality analyses, particularly if the length of follow-up time is <6 years.

3. Can the authors explain why they chose to explore the association between sedentary behavior and cancer-related mortality outcomes in a sample of cancer-free adults, rather than a cancer population? Did the authors also explore all-cause mortality?

4. To the above point, are the authors able to comment as to whether PA and SB behaviors were measured prior to or after a diagnosis of cancer, which preceded the mortality outcome? This is an important consideration when messaging about lifestyle behavior change.

Any attachments provided with reviews can be seen via the following link: [LINK]

--------------------------------------------------------- ---

General editorial requests:

(Note: not all will apply to your paper, but please check each item carefully)

*We ask every co-author listed on the manuscript to fill in a contributing author statement, making sure to declare all competing interests. If any of the co-authors have not filled in the statement, we will remind them to do so when the paper is revised. If all statements are not completed in a timely fashion this could hold up the re-review process. If new competing interests are declared later in the revision process, this may also hold up the submission. Should there be a problem getting one of your co-authors to fill in a statement we will be in contact. Please do not add or remove authors without first discussing this with the handling editor. You can see our competing interests policy here: http://journals.plos.org/plosmedicine/s/competing-interests.

*Please upload any figures associated with your paper as individual TIF or EPS files with 300dpi resolution at resubmission; please read our figure guidelines for more information on our requirements: http://journals.plos.org/plosmedicine/s/figures. While revising your submission, we strongly recommend that you use PLOS's NAAS tool (https://ngplosjournals.pagemajik.ai/artanalysis) to test your figure files. NAAS can convert your figure files to the TIFF file type and meet basic requirements (such as print size, resolution), or provide you with a report on issues that do not meet our requirements and that NAAS cannot fix.

After uploading your figures to PLOS's NAAS tool - https://ngplosjournals.pagemajik.ai/artanalysis, NAAS will process the files provided and display the results in the "Uploaded Files" section of the page as the processing is complete.

If the uploaded figures meet our requirements (or NAAS is able to fix the files to meet our requirements), the figure will be marked as "fixed" above. If NAAS is unable to fix the files, a red "failed" label will appear above.

When NAAS has confirmed that the figure files meet our requirements, please download the file via the download option, and include these NAAS processed figure files when submitting your revised manuscript.

*Please ensure that the paper adheres to the PLOS Data Availability Policy (see http://journals.plos.org/plosmedicine/s/data-availability), which requires that all data underlying the study's findings be provided in a repository or as Supporting Information. For data residing with a third party, authors are required to provide instructions with contact information (web or email address) for obtaining the data. Please note that a study author cannot be the contact person for the data. PLOS journals do not allow statements supported by "data not shown" or "unpublished results." For such statements, authors must provide supporting data or cite public sources that include it.

*We expect all researchers with submissions to PLOS in which author-generated code underpins the findings in the manuscript to make all author-generated code available without restrictions upon publication of the work. In cases where code is central to the manuscript, we may require the code to be made available as a condition of publication. Authors are responsible for ensuring that the code is reusable and well documented. Please make any custom code available, either as part of your data deposition or as a supplementary file. Please add a sentence to your data availability statement regarding any code used in the study, e.g. "The code used in the analysis is available from Github [URL] and archived in Zenodo [DOI link]" Please review our guidelines at https://journals.plos.org/plosmedicine/s/materials-software-and-code-sharing and ensure that your code is shared in a way that follows best practice and facilitates reproducibility and reuse. Because Github depositions can be readily changed or deleted, we encourage you to make a permanent DOI'd copy (e.g. in Zenodo) and provide the URL.

FORMATTING - GENERAL

*Abstract: Please structure your abstract using the PLOS Medicine headings (Background, Methods and Findings, Conclusions). Please combine the Methods and Findings sections into one section.

*At this stage, we ask that you include a short, non-technical Author Summary of your research to make findings accessible to a wide audience that includes both scientists and non-scientists. The Author Summary should immediately follow the Abstract in your revised manuscript. This text is subject to editorial change and should be distinct from the scientific abstract. Ideally each sub-heading should contain 2-3 single sentence, concise bullet points containing the most salient points from your study. In the final bullet point of 'What Do These Findings Mean?', please include the main limitations of the study in non-technical language. Please see our author guidelines for more information: https://journals.plos.org/plosmedicine/s/revising-your-manuscript#loc-author-summary.

*Please express the main results with 95% CIs as well as p values. When reporting p values please report as p<0.001 and where higher as the exact p value p=0.002, for example. Throughout, suggest reporting statistical information as follows to improve clarity for the reader "22% (95% CI [13%,28%]; p</=)". Please be sure to define all numerical values at first use.

*Please include page numbers and line numbers in the manuscript file. Use continuous line numbers (do not restart the numbering on each page).

*Please cite the reference numbers in square brackets. Citations should precede punctuation.

FIGURES AND TABLES

*Please provide titles and legends for all figures and tables (including those in Supporting Information files).

*Please define all abbreviations used in each figure/table (including those in Supporting Information files).

*Please consider avoiding the use of red and green in order to make your figure more accessible to those with color blindness.

SUPPLEMENTARY MATERIAL

*Please note that supplementary material will be posted as supplied by the authors. Therefore, please amend it according to the relevant comments outlined here.

*Please cite your Supporting Information as outlined here: https://journals.plos.org/plosmedicine/s/supporting-information

REFERENCES

*PLOS uses the numbered citation (citation-sequence) method and first six authors, et al.

*Please ensure that journal name abbreviations match those found in the National Center for Biotechnology Information (NCBI) databases (http://www.ncbi.nlm.nih.gov/nlmcatalog/journals), and are appropriately formatted and capitalised.

*Where website addresses are cited, please include the complete URL and specify the date of access (e.g. [accessed: 12/06/2023]).

*Please also see https://journals.plos.org/plosmedicine/s/submission-guidelines#loc-references for further details on reference formatting.

OBSERVATIONAL STUDIES

*Abstract: Please include the study design, population and setting, number of participants, years during which the study took place (enrollment and follow up), length of follow up, and main outcome measures.

*Please ensure that the study is reported according to the STROBE (or appropriate STOBE extension) guideline (available from: https://www.equator-network.org/reporting-guidelines/strobe) and include the completed STROBE (or STROBE extension) checklist as Supporting Information. Please add the following statement, or similar, to the Methods: "This study is reported as per the Strengthening the Reporting of Observational Studies in Epidemiology (STROBE) guideline (S1 Checklist)." When completing the checklist, please use section and paragraph numbers, rather than page numbers.

*Please ensure that the study is reported according to the RECORD guideline (available from https://www.record-statement.org) and include the completed checklist as Supporting Information. Please add the following statement, or similar, to the Methods: "This study is reported as per the Reporting of Studies Conducted using Observational Routinely-Collected Data (RECORD) guideline (S1 Checklist)." When completing the checklist, please use section and paragraph numbers, rather than page numbers.

*Please ensure that the study is reported according to the GATHER statement (available from https://www.equator-network.org/reporting-guidelines/gather-statement) and include the completed checklist as Supporting Information. Please add the following statement, or similar, to the Methods: "This study is reported as per the Guidelines for Accurate and Transparent Health Estimates Reporting (GATHER) statement (S1 Checklist)." When completing the checklist, please use section and paragraph numbers, rather than page numbers.

*We recommend that the study is reported according to the AGReMA statement (https://agrema-statement.org/#:~:text=AGReMA%20is%20an%20evidence%2D%20and,randomised%20trials%20and%20observational%20studies) and include the completed checklist as Supporting Information. Please add the following statement, or similar, to the Methods: "This study is reported as per the Guideline for Reporting Mediation Analyses (AGReMA) statement (S1 Checklist)." When completing the checklist, please use section and paragraph numbers, rather than page numbers.

*For all observational studies, in the manuscript text, please indicate: (1) the specific hypotheses you intended to test, (2) the analytical methods by which you planned to test them, (3) the analyses you actually performed, and (4) when reported analyses differ from those that were planned, transparent explanations for differences that affect the reliability of the study's results. If a reported analysis was performed based on an interesting but unanticipated pattern in the data, please be clear that the analysis was data driven.

*Please state in the Methods section whether the study had a prospective protocol or analysis plan. If a prospective analysis plan (from your funding proposal, IRB or other ethics committee submission, study protocol, or other planning document written before analyzing the data) was used in designing the study, please include the relevant document(s) with your revised manuscript as a Supporting Information file to be published alongside your study and cite it in the Methods section. A legend for this file should be included at the end of your manuscript. If no such document exists, please make sure that the Methods section transparently describes when analyses were planned, and when/why any data-driven changes to analyses took place. Changes in the analysis, including those made in response to peer review comments, should be identified as such in the Methods section of the paper, with rationale.

MODELLING STUDIES

The following list is derived from Geoffrey P Garnett, Simon Cousens, Timothy B Hallett, Richard Steketee, Neff Walker. Mathematical models in the evaluation of health programmes. (2011) Lancet DOI:10.1016/S0140-6736(10)61505-X:

*If pertinent, please provide a diagram that shows the model structure, including how the natural history of the disease is represented, the process and determinants of disease acquisition, and how the putative intervention could affect the system.

*Please provide a complete list of model parameters, including clear and precise descriptions of the meaning of each parameter, together with the values or ranges for each, with justification or the primary source cited and important caveats about the use of these values noted.

*Please provide a clear statement about how the model was fitted to the data, including goodness-of-fit measure, the numerical algorithm used, which parameter varied, constraints imposed on parameter values, and starting conditions.

*For uncertainty analyses, please state the sources of uncertainties quantified and not quantified [can include parameter, data, and model structure].

*Please provide sensitivity analyses to identify which parameter values are most important in the model. Uncertainty estimates seek to derive a range of credible results on the basis of an exploration of the range of reasonable parameter values. The choice of method should be presented and justified.

*Please discuss the scientific rationale for the choice of model structure and identify points where this choice could influence conclusions drawn. Please also describe the strength of the scientific basis underlying the key model assumptions.

*For studies that develop a prediction model or evaluate its performance, please ensure that the study is reported according to the TRIPOD statement (https://www.equator-network.org/reporting-guidelines/tripod-statement) and include the completed checklist as Supporting Information. Please add the following statement, or similar, to the Methods: "This study is reported as per the Transparent Reporting of a Multivariable Prediction Model for Individual Prognosis Or Diagnosis (TRIPOD) statement (S1 Checklist)." For studies using machine learning, please use the TRIPOD-AI checklist. When completing the checklist, please use section and paragraph numbers, rather than page numbers.

# Author comment: Author response to Decision Letter 2

**DOI:** 10.1371/journal.pmed.1004767.r003

1 Apr 2026

Attachment Submitted filename: Response letter_plos med.docx

# Peer review: Decision Letter 2

**DOI:** 10.1371/journal.pmed.1004767.r004

**Authors:** Jennifer Thorley, Andreia Cunha, Andreia Cunha

12 May 2026

Dear Dr. Ho,

Apologies for the delay in getting back to you with a decision, which was due to challenges in securing all the necessary advice. Thank you very much for re-submitting your manuscript "Accelerometry-measured prolonged and interrupted sedentary behavior and cancer incidence and mortality: A prospective cohort study of 92,354 UK Biobank participants" (PMEDICINE-D-25-03223R2) for review by PLOS Medicine.

I have discussed the paper with my colleagues and the academic editor and it was also seen again by two original reviewers. The academic editor also checked your response to the reviewer who was unavailable. I am pleased to say that provided the remaining editorial and production issues are dealt with we are planning to accept the paper for publication in the journal.

The remaining issues that need to be addressed are listed at the end of this email. Any accompanying reviewer attachments can be seen via the link below. Please take these into account before resubmitting your manuscript:

[LINK]

***Please note while forming your response, if your article is accepted, you may have the opportunity to make the peer review history publicly available. The record will include editor decision letters (with reviews) and your responses to reviewer comments. If eligible, we will contact you to opt in or out.***

In revising the manuscript for further consideration here, please ensure you address the specific points made by each reviewer and the editors. In your rebuttal letter you should indicate your response to the reviewers' and editors' comments and the changes you have made in the manuscript. Please submit a clean version of the paper as the main article file. A version with changes marked must also be uploaded as a marked up manuscript file.

Please also check the guidelines for revised papers at http://journals.plos.org/plosmedicine/s/revising-your-manuscript for any that apply to your paper. If you haven't already, we ask that you provide a short, non-technical Author Summary of your research to make findings accessible to a wide audience that includes both scientists and non-scientists. The Author Summary should immediately follow the Abstract in your revised manuscript. This text is subject to editorial change and should be distinct from the scientific abstract.

In addition to these revisions, you may need to complete some formatting changes, which you will receive in a follow up email. A member of our team will be in touch with a set of requests shortly. If you do not receive a separate email within a few days, please assume that checks have been completed, and no additional changes are required.

We expect to receive your revised manuscript within 1 week. Please email us (plosmedicine@plos.org) if you have any questions or concerns.

We ask every co-author listed on the manuscript to fill in a contributing author statement. If any of the co-authors have not filled in the statement, we will remind them to do so when the paper is revised. If all statements are not completed in a timely fashion this could hold up the re-review process. Should there be a problem getting one of your co-authors to fill in a statement we will be in contact. YOU MUST NOT ADD OR REMOVE AUTHORS UNLESS YOU HAVE ALERTED THE EDITOR HANDLING THE MANUSCRIPT TO THE CHANGE AND THEY SPECIFICALLY HAVE AGREED TO IT.

Please ensure that the paper adheres to the PLOS Data Availability Policy (see http://journals.plos.org/plosmedicine/s/data-availability), which requires that all data underlying the study's findings be provided in a repository or as Supporting Information. For data residing with a third party, authors are required to provide instructions with contact information for obtaining the data. PLOS journals do not allow statements supported by "data not shown" or "unpublished results." For such statements, authors must provide supporting data or cite public sources that include it.

To enhance the reproducibility of your results, we recommend that you deposit your laboratory protocols in protocols.io, where a protocol can be assigned its own identifier (DOI) such that it can be cited independently in the future. Additionally, PLOS ONE offers an option to publish peer-reviewed clinical study protocols. Read more information on sharing protocols at https://plos.org/protocols?utm_medium=editorial-email&utm_source=authorletters&utm_campaign=protocols

Please review your reference list to ensure that it is complete and correct. If you have cited papers that have been retracted, please include the rationale for doing so in the manuscript text, or remove these references and replace them with relevant current references. Any changes to the reference list should be mentioned in the rebuttal letter that accompanies your revised manuscript.

Please note, when your manuscript is accepted, an uncorrected proof of your manuscript will be published online ahead of the final version, unless you've already opted out via the online submission form. If, for any reason, you do not want an earlier version of your manuscript published online or are unsure if you have already indicated as such, please let the journal staff know immediately at plosmedicine@plos.org.

If you have any questions in the meantime, please contact me or the journal staff on plosmedicine@plos.org.

We look forward to receiving the revised manuscript by May 19 2026 11:59PM.

Sincerely,

Andreia Cunha, PhD

Senior Editor

PLOS Medicine

plosmedicine.org

------------------------------------------------------------

Requests from Editors:

GENERAL EDITORIAL REQUESTS

* Please confirm that your title complies with PLOS Medicine's style. Your title must be nondeclarative and not a question. It should begin with main concept if possible. "Effect of" should be used only if causality can be inferred, i.e., for an RCT. Please place the study design ("A randomized controlled trial," "A retrospective study," "A modelling study," etc.) in the subtitle (ie, after a colon).

* Please confirm that your abstract complies with our requirements, including format (three sections: Background, Methods and Findings, and Conclusions) and providing all the information relevant to this study type https://journals.plos.org/plosmedicine/s/submission-guidelines#loc-abstract

* In the abstract, please include the important dependent variables that are adjusted for in the analyses.

* In the last sentence of the Abstract Methods and Findings section, please describe the main limitation(s) of the study's methodology.

* In the author summary, in the final bullet point of 'What Do These Findings Mean?', please include the main limitations of the study in non-technical language. Please see our author guidelines for more information: https://journals.plos.org/plosmedicine/s/revising-your-manuscript#loc-author-summary."

* Please ensure that the Introduction ends with a clear description of the study question or hypothesis.

* Statistical reporting: Please revise throughout the manuscript, including tables and figures.

- Please report statistical information as follows to improve clarity for the reader 22% (95% CI [13,28]; p</=).

- Please separate upper and lower bounds with commas instead of hyphens as the latter can be confused with reporting of negative values.

- Please repeat statistical definitions (HR, CI etc.) for each set of parentheses.

* Please remove the 'conclusions' subheading from the discussion. Please also remove any other subheadings from the discussion.

* Please ensure that all abbreviations are defined at first use throughout the text.

* Please confirm that all numbers presented in the abstract are present and identical to numbers presented in the main manuscript text.

FUNDING STATEMENT

* The funding statement should include: specific grant numbers, initials of authors who received each award, URLs to sponsors’ websites. Also, please state whether any sponsors or funders (other than the named authors) played any role in study design, data collection and analysis, the decision to publish, or preparation of the manuscript. If they had no role in the research, include this sentence: “The funders had no role in study design, data collection and analysis, decision to publish, or preparation of the manuscript.”

COMPETING INTERESTS STATEMENT

* All authors must declare their relevant competing interests per the PLOS policy, which can be seen here: https://journals.plos.org/plosmedicine/s/competing-interests For authors with ties to industry, please indicate whether any of the interests has a financial stake in the results of the current study.

* Please add this statement to the manuscript's Competing Interests: "[Initials] is a paid statistical consultant on PLOS Medicine's statistical board."

DATA AVAILABILITY

* PLOS defines the “minimal data set” to consist of the data set used to reach the conclusions drawn in the manuscript with related metadata and methods, and any additional data required to replicate the reported study findings in their entirety. Authors do not need to submit their entire data set, or the raw data collected during an investigation. Please submit the following data:

The values behind the means, standard deviations and other measures reported;

The values used to build graphs;

The points extracted from images for analysis.

* The Data Availability Statement (DAS) requires revision. If the data are not freely available, please describe briefly the ethical, legal, or contractual restriction that prevents you from sharing it. Please also include an appropriate contact (web or email address) for inquiries (again, this cannot be a study author).

ETHICS AND CONSENT

* Please move the following sentence from the acknowledgements ethics section and clarify in the statement f the numbers provided refer to approval for data use: 'This research has been conducted using the UK Biobank Resource under Application Number 7155 and 46466.'

FIGURES

* Please consider moving the flow diagram shared in the supplementary information as Figure 1 into the main text to aid the reader.

* Please provide titles and legends for all figures and tables (including those in Supporting Information files). Please define all acronyms used in each figure or table in its corresponding legend.

* Please ensure that where relevant figures include 95% CIs.

* Please consider avoiding the use of green in order to make your figure more accessible

* Please provide the unadjusted comparisons as well as the adjusted comparisons in all relevant Tables

* Please specify the variables controlled for in all relevant Tables

OBSERVATIONAL, COHORT, CROSS-SECTIONAL, AND CASE CONTROL STUDIES

* Please ensure that the study is reported according to the STROBE guideline, and include the completed STROBE checklist as Supporting Information. Please add the following statement, or similar, to the Methods: ""This study is reported as per the Strengthening the Reporting of Observational Studies in Epidemiology (STROBE) guideline (S1 Checklist).""

The STROBE guideline can be found here: http://www.equator-network.org/reporting-guidelines/strobe/

When completing the checklist, please use section and paragraph numbers, rather than page numbers.

* Did your study have a prospective protocol or analysis plan? Please state this (either way) early in the Methods section.

a) If a prospective analysis plan (from your funding proposal, IRB or other ethics committee submission, study protocol, or other planning document written before analyzing the data) was used in designing the study, please include the relevant prospectively written document with your revised manuscript as a Supporting Information file to be published alongside your study, and cite it in the Methods section. A legend for this file should be included at the end of your manuscript.

b) If no such document exists, please make sure that the Methods section transparently describes when analyses were planned, and when/why any data-driven changes to analyses took place.

c) In either case, changes in the analysis-- including those made in response to peer review comments-- should be identified as such in the Methods section of the paper, with rationale."

* Your study is observational and therefore causality cannot be inferred. Please remove language that implies causality and refer to associations instead.

* For all observational studies, in the manuscript text, please indicate: (1) the specific hypotheses you intended to test, (2) the analytical methods by which you planned to test them, (3) the analyses you actually performed, and (4) when reported analyses differ from those that were planned, transparent explanations for differences that affect the reliability of the study's results. If a reported analysis was performed based on an interesting but unanticipated pattern in the data, please be clear that the analysis was data-driven.

Comments from Reviewers:

Reviewer #1: All my comments have been addressed

Reviewer #3: The authors were comprehensive in their responses to the reviewers and sufficiently addressed the comments. The resulting manuscript has been strengthened by the revisions.

Any attachments provided with reviews can be seen via the following link:

[LINK]

# Author comment: Author response to Decision Letter 3

**DOI:** 10.1371/journal.pmed.1004767.r005

27 May 2026

Attachment Submitted filename: minor revision_response letter.docx

# Editor report: Decision Letter 3

**DOI:** 10.1371/journal.pmed.1004767.r006

**Authors:** Jennifer Thorley, Andreia Cunha, Andreia Cunha, Andreia Cunha

29 May 2026

Dear Dr Ho,

On behalf of my colleagues and the Academic Editor, Steven Moore, I am pleased to inform you that we have agreed to publish your manuscript "Accelerometry-measured prolonged and interrupted sedentary behavior and cancer incidence and mortality: A prospective cohort study of 92,354 UK Biobank participants" (PMEDICINE-D-25-03223R3) in PLOS Medicine.

Before your manuscript can be formally accepted you will need to complete some formatting changes, which you will receive in a follow up email. Please be aware that it may take several days for you to receive this email; during this time no action is required by you. Once you have received these formatting requests, please note that your manuscript will not be scheduled for publication until you have made the required changes.

In the meantime, please log into Editorial Manager at http://www.editorialmanager.com/pmedicine/, click the "Update My Information" link at the top of the page, and update your user information to ensure an efficient production process.

PRESS

We frequently collaborate with press offices. If your institution or institutions have a press office, please notify them about your upcoming paper at this point, to enable them to help maximise its impact. If the press office is planning to promote your findings, we would be grateful if they could coordinate with medicinepress@plos.org. If you have not yet opted out of the early version process, we ask that you notify us immediately of any press plans so that we may do so on your behalf.

We also ask that you take this opportunity to read our Embargo Policy regarding the discussion, promotion and media coverage of work that is yet to be published by PLOS. As your manuscript is not yet published, it is bound by the conditions of our Embargo Policy. Please be aware that this policy is in place both to ensure that any press coverage of your article is fully substantiated and to provide a direct link between such coverage and the published work. For full details of our Embargo Policy, please visit http://www.plos.org/about/media-inquiries/embargo-policy/.

To enhance the reproducibility of your results, we recommend that you deposit your laboratory protocols in protocols.io, where a protocol can be assigned its own identifier (DOI) such that it can be cited independently in the future. Additionally, PLOS ONE offers an option to publish peer-reviewed clinical study protocols. Read more information on sharing protocols at https://plos.org/protocols?utm_medium=editorial-email&utm_source=authorletters&utm_campaign=protocols

Thank you again for submitting to PLOS Medicine. We look forward to publishing your paper.

Sincerely,

Andreia Cunha, PhD

Senior Editor

PLOS Medicine
